# Elucidating the Novel Mechanism of Ligustrazine in Preventing Postoperative Peritoneal Adhesion Formation

**DOI:** 10.1155/2022/9226022

**Published:** 2022-03-10

**Authors:** Lili Yang, Zhengjun Li, Yao Chen, Feiyan Chen, Haopeng Sun, Min Zhao, Yanqi Chen, Yali Wang, Wenlin Li, Li Zeng, Yaoyao Bian

**Affiliations:** ^1^Jiangsu Provincial Engineering Center of TCM External Medication Researching and Industrializing, Nanjing University of Chinese Medicine, Nanjing 210023, China; ^2^Jingwen Library, Nanjing University of Chinese Medicine, Nanjing 210023, China; ^3^School of First Clinical Medicine, Nanjing University of Chinese Medicine, Nanjing 210023, China; ^4^College of Health Economics Management, Nanjing University of Chinese Medicine, Nanjing 210023, China; ^5^School of Pharmacy, Nanjing University of Chinese Medicine, Nanjing 210023, China; ^6^School of Preclinical Medicine, Nanjing University of Chinese Medicine, Nanjing 210023, China; ^7^Department of Medicinal Chemistry, China Pharmaceutical University, Nanjing 211198, China; ^8^School of Traditional Chinese Medicine, School of Integrated Chinese and Western Medicine, Nanjing University of Chinese Medicine, Nanjing 210023, China; ^9^College of Acupuncture and Massage, College of Regimen and Rehabilitation, Nanjing University of Chinese Medicine, Nanjing 210023, China

## Abstract

Postoperative peritoneal adhesion (PPA) is a major clinical complication after open surgery or laparoscopic procedure. Ligustrazine is the active ingredient extracted from the natural herb *Ligusticum chuanxiong Hort*, which has promising antiadhesion properties. This study is aimed at revealing the underlying mechanisms of ligustrazine in preventing PPA at molecular and cellular levels. Both rat primary peritoneal mesothelial cells (PMCs) and human PMCs were used for analysis in vitro. Several molecular biological techniques were applied to uncover the potential mechanisms of ligustrazine in preventing PPA. And molecular docking and site-directed mutagenesis assay were used to predict the binding sites of ligustrazine with PPAR*γ*. The bioinformatics analysis was further applied to identify the key pathway in the pathogenesis of PPA. Besides, PPA rodent models were prepared and developed to evaluate the novel ligustrazine nanoparticles *in vivo*. Ligustrazine could significantly suppress hypoxia-induced PMC functions, such as restricting the production of profibrotic cytokines, inhibiting the expression of migration and adhesion-associated molecules, repressing the expression of cytoskeleton proteins, restricting hypoxia-induced PMCs to obtain myofibroblast-like phenotypes, and reversing ECM remodeling and EMT phenotype transitions by activating PPAR*γ*. The antagonist GW9662 of PPAR*γ* could restore the inhibitory effects of ligustrazine on hypoxia-induced PMC functions. The inhibitor KC7F2 of HIF-1*α* could repress hypoxia-induced PMC functions, and ligustrazine could downregulate the expression of HIF-1*α*, which could be reversed by GW9662. And the expression of HIF-1*α* inhibited by ligustrazine was dramatically reversed after transfection with si-SMRT. The results showed that the benefit of ligustrazine on PMC functions is contributed to the activation of PPAR*γ* on the transrepression of HIF-1*α* in an SMRT-dependent manner. Molecular docking and site-directed mutagenesis tests uncovered that ligustrazine bound directly to PPAR*γ*, and Val 339/Ile 341 residue was critical for the binding of PPAR*γ* to ligustrazine. Besides, we discovered a novel nanoparticle agent with sustained release behavior, drug delivery efficiency, and good tissue penetration in PPA rodent models. Our study unravels a novel mechanism of ligustrazine in preventing PPA. The findings indicated that ligustrazine is a potential strategy for PPA formation and ligustrazine nanoparticles are promising agents for preclinical application.

## 1. Introduction

Postoperative peritoneal adhesion (PPA) is a major clinical complication after open surgery or laparoscopic procedure. It may cause a range of complications including acute bowel obstruction and chronic adhesion symptoms, such as abdominal pain or female infertility [1]. PPA may increase the subsequent reoperation rate, prolong the time of hospitalization, and increase medical costs. A recent retrospective cohort study performed in Scotland showed that 72,270 (17.6%) surgical patients among 12,687 patients who were readmitted within 5 years for adhesion-related problems were possibly associated with adhesions, 9436 (13.1%) were potentially complicated by adhesion, and 2527 (3.5%) were directly related to adhesion [2]. And PPA has brought heavy economic impacts on individuals, families, and the whole society [3].

Peritoneal mesothelial cells (PMCs), as the main cells of the functional peritoneum, are involved in the pathogenesis of adhesion formation [4]. Injured PMCs can induce profibrotic cytokine expression [5], i.e., vascular endothelial growth factor (VEGF) and connective tissue growth factor (CTGF), which can lead to inflammatory response and even result in peritoneal adhesion [6]. Existing studies [7, 8] found that hypoxia caused by tissue injury is the most important factor in adhesion formation. Under the oxygen-deficient condition, adhesion molecules elevated, cytoskeleton reorganized, epithelial markers lost, and mesenchymal markers acquired, leading to excess accumulation of extracellular matrix (ECM) and epithelial-mesenchymal transition (EMT) [9, 10]. However, the exact pathogenesis of hypoxia-induced PMC driving adhesion formation is precisely unclear. Besides, with the development of material science and life science, mounting barriers or biological materials and antiadhesion drugs have gained much attention in adhesion prevention. Each material or drug has its advantages and disadvantages [11–13]. Hence, it is also urgent to identify novel and more effective agents in preventing PPA.

Fortunately, nanoparticle application with unique advantages is considered as a promising approach in disease prevention and treatment, such as postoperative adhesions [14] and peritoneal fibrosis [15]. Intriguingly, our previous study confirmed that the bioactive alkaloid ligustrazine has significant effectiveness in preventing adhesion formation [16, 17]. Ligustrazine is the active ingredient that is extracted from the root of natural herbal *Ligusticum chuanxiong Hort* (Umbelliferae). It has a hot spot in the field of cardiovascular, tumor, and other diseases [18]. Recently, ligustrazine had also attracted considerable attention in the inflammatory response [19]. Hence, we hypothesized that there might be a close relation between ligustrazine and the pathological EMT process in hypoxia-induced PMCs. In this study, we set out to explore the underlying molecular mechanisms of ligustrazine in preventing PPA both *in vivo* and *in vitro*. Our findings might generate new sights into the therapeutic strategies for PPA and good prospects for the preclinical application of ligustrazine nanoparticles (LN).

## 2. Material and Methods

### 2.1. Chemicals and Antibodies

Ligustrazine (C_8_H_12_N_2_, purity > 98%) was obtained from Tokyo Chemical Industry (Japan). GW9662 (C_13_H_9_ClN_2_O_3_) and KC7F2 (C_16_H_16_Cl_4_N_2_O_4_S_4_) were purchased from Selleck (USA). The above chemicals were dissolved in dimethyl sulfoxide (DMSO) (Sigma-Aldrich, USA) according to different ratios. The vehicle control group was only treated with DMSO. VEGF (sc-57496, Santa Cruz, USA), CTGF (sc-373936, Santa Cruz, USA), vascular cell adhesion molecule-1 (VCAM-1; ab134047, Abcam, USA), intercellular cell adhesion molecular-1 (ICAM-1; sc-8439, Santa Cruz, USA), cofilin (bs-2759R, Bioss, China), phospho-cofilin (p-cofilin, bs-20261R, Bioss, China), fibroblast-specific protein 1 (FSP1; bs-3759R, Bioss, China), matrix metalloproteinases 2 (MMP2; bs-4605R, Bioss, China), tissue inhibitor of metalloproteinases-1 (TIMP-1; bs-0415R, Bioss, China), E-cadherin (bs-1519R, Bioss, China), cytokeratin 18 (bs-2043R, Bioss, China), Snail (bs-1371R, Bioss, China), *α*-smooth muscle actin (*α*-SMA; bs-10196R, Bioss, China), F-actin (FITC-conjugated phalloidin, C1033, Beyotime, China), vinculin (bs-6640R, Bioss, China), peroxisome proliferator-activated receptor *γ* (PPAR*γ*; bs-4590R, Bioss, China), lamin B1 (sc-377000, Santa Cruz, USA), *β*-actin (CW0096M, CWBIO, China), and hypoxia-inducible factor 1*α* (HIF-1*α*; sc-71247, Santa Cruz, USA) were used. RPMI 1640 medium (Hyclone, USA), fetal bovine serum (EVERY GREEN, China), and trypsin/EDTA (Gibco, USA) were provided. Lipofectamine 3000 (L3000008) was obtained from Invitrogen (USA). Second antibodies such as anti-rabbit IgG (Cy3, CW0159S, CWBIO, China; FITC, CW0114S, CWBIO, China) and anti-mouse IgG (Cy3, CW0145S, CWBIO, China; FITC, A0568, Beyotime, China) were provided.

### 2.2. Cell Culture

Rat primary PMCs (RPMCs) were isolated based on previous studies [17, 20]. Briefly, after the Sprague-Dawley (SD) rats were sacrificed with cervical dislocation, the abdominal skin was prepared and sterilized under aseptic conditions. 20 ml 0.25% trypsin-0.016% EDTA was injected into the peritoneal cavity for 20 min. During this period, the abdomen of the rats could be kneaded, and then, 10 ml complete medium with 10% FBS was injected into the abdominal cavity to end the digestion. The peritoneal fluid was collected, followed by centrifugation at 150 × *g* for 10 min. Cells were suspended and cultured in RPMI 1640 medium containing 10% FBS, 1% penicillin, and streptomycin in the incubator under 5% CO_2_ at 37°C. The third-generation cells were used for the following research as previously described [17]. Besides, human PMCs (HPMCs and HMrSV5) were donated by Professor Sheng's team of the first clinical medical college in Nanjing University of Chinese Medicine. HPMCs were cultured as above. To induce hypoxia, PMCs were seeded under hypoxic conditions, which were maintained by pumping a mixture of gases (1% O_2_, 94% N_2_, and 5% CO_2_) into a hypoxia chamber at 37°C.

### 2.3. Small Interfering RNA (siRNA) Transfection Assay

HPMCs were seeded at 6 × 10^5^ cells per well onto 6-well plates. When the cell density reached 60%, cells were transfected with equivalent amounts of siRNA targeting silencing mediator of retinoid and thyroid receptor (SMRT; sc-36514, Santa Cruz, USA), nuclear receptor coexpression (NCoR; sc-36001, Santa Cruz, USA), or negative control siRNA (sc-37007, Santa Cruz, USA) in serum and antibiotics-free medium for 24 h by using Lipofectamine 3000 according to the manufacturer's instructions. Then, a quantitative real-time polymerase chain reaction (qRT-PCR) was used to determine infection efficiency.

### 2.4. Enzyme-Linked Immunosorbent Assays (ELISA)

RPMCs or HPMCs were seeded with different concentrations of ligustrazine for 24 h. The levels of VEGF and CTGF in the supernatants were detected by ELISA kits (Jinyibai, China) according to the manufacturer's instructions. The OD values of each well were measured at the wavelength of 450 nm under the enzymatic analyzer.

### 2.5. Cell Adhesion Assays

Before RPMCs or HPMCs were seeded at 2 × 10^5^ cells per well in 24-well plates, each well was covered with 200 *μ*l fibronectin at 37°C for 2 h. The cells were treated with vehicle or ligustrazine at different concentrations for 24 h. After removing unattached cells and the old culture medium, the plates were washed twice with phosphate-buffered saline (PBS). The attached cells were cultured with 5 *μ*g/ml fluorescein diacetate (FDA; F8040, Solarbio, China)/acetone solution at 37°C for 10 min. Then, cells were washed twice with PBS. Finally, cells in each well were imaged randomly under the fluorescence microscope (Nikon, Ti, Japan), and the number of adherent cells in each field was calculated by the naked eyes. The representative pictures were presented.

### 2.6. Cell Proliferation Assays

RPMCs or HPMCs were plated at 4 × 10^4^ cells per well in 96-well plates, respectively. When grown to 50-60% confluence, cells were synchronized in a serum-free medium for 24 h and cultured in a complete medium with vehicle or ligustrazine at different concentrations for another 24 h. Then, cells were incubated with MTT (Beyotime, C0009S, China) for 2 h at 37°C. The absorbance was observed by setting the OD value at 570 nm using the analyzer.

### 2.7. Flow Cytometry Analysis of Nuclear PPAR*γ* Translocation

RPMCs or HPMCs were treated with vehicle or ligustrazine at different concentrations for another 24 h after synchronization in the serum-free medium for 24 h. When the cell density reached 70-80%, the cells were trypsinized and collected about 1 × 10^6^ per group. After fixation with a fixed buffer, the cells were incubated with the PPAR*γ* antibody away from light at 37°C for 1 h, followed by incubation with FITC-labeled goat anti-rabbit IgG for another 1 h at room temperature. Then, the nuclear was stained by 7-AAD for 5 min. Finally, the nuclear PPAR*γ* translocation was determined by flow cytometry (Amnis, Millipore, USA). The representative views were shown.

### 2.8. Flow Cytometry Analysis of Intracellular Ca^2+^ Levels

Cell processing and harvest methods were mentioned as described above. Cells were incubated with 1 *μ*g/ml fluo-3 acetoxymethyl (fluo-3AM; S1056, Beyotime, China), a kind of fluorescent ratiometric calcium indicator for 1 h at room temperature to ensure that the intracellular Flo-3M changed into fluo-3. Then, cells were washed three times with PBS and centrifuged at 4°C for 15 min. Finally, the fluorescence intensity of fluo-3, represented as the levels of intracellular Ca^2+^, was analyzed at the wavelength of 488 nm by flow cytometry (Amnis, Millipore, USA). The representative histogram graphs were shown.

### 2.9. Cytoskeleton Staining

RPMCs or HPMCs were seeded in 6-well plates and treated with vehicle or ligustrazine at different concentrations for 24 h when the cell density reached 50%. After removing the old culture medium, the cells were fixed with PBS containing 3.7% paraformaldehyde for 10 min and then washed three times with PBS containing 0.1% Triton X-100 for 5 min each time at 37°C. Cells were incubated with Actin-Tracker Green (C1033, Beyotime, China) in dark conditions for 60 min following the instructions. Then, cells were washed and stained by diamidino-phenyl-indole (DAPI) for 5 min. After three washes with PBS, random sections of the cells were imaged under the fluorescence microscope (Nikon, Ti, Japan) and representative graphs were shown.

### 2.10. Molecular Docking

The structure of ligustrazine was drawn in ChemBioDraw Ultra 14.0, saved as mol format, and then prepared in Discovery Studio 3.0 (DS 3.0) and minimized by 2000 steps of the steepest descent method followed by 2000 steps of conjugate gradient method. The 3D structure of receptor protein was downloaded from the protein data bank (PDB ID: 6DGO) and prepared by using the “prepare protein” module in DS 3.0 with default parameters. The molecular docking simulation was performed by the CDOCKER module in DS 3.0. The binding site was defined by the origin ligand GD4 in a radius of 10 Å. Other parameters were kept as default. The intermolecular interactions were performed by using the PyMOL Molecular Graphics System (Version 2.2.2).

### 2.11. Site-Directed Mutagenesis Based on Overlap Extension PCR

The primers of PPAR*γ* wild type (WT) or PPAR*γ* mutant type (MT) were synthesized by GenScript (Nanjing, China), as presented in Supplementary Table S1. cDNA of human cardiomyocyte cells was used as a template as previously described [21]. Homologous recombination was used to subclone the amplicon into pET-28a to generate pET10. Using site-directed mutagenesis with pET10 as a template, pET11, pET12, pET13, pET14, and pET15 were constructed, which have mutations of glutamine at position 318 to proline (Cys 285/Arg 288, mutant 1) (Supplementary Figure S1), arginine at position 320 to threonine (Leu 330/Leu 333, mutant 2) (Supplementary Figure S2), serine at position 285 to threonine (Val 339/Ile 341, mutant 3) (Supplementary Figure S3), serine at position 285 to threonine (Met 348, mutant 4) (Supplementary Figure S4), and serine at position 285 to threonine (Met 364, mutant 5) (Supplementary Figure S5), respectively.

### 2.12. Protein Overexpression and Purification

The recombinant His-tagged PPAR*γ* was expressed as previously described [21]. Briefly, the plasmids (pET10, pET11, pET12, pET13, pET14, or pET15) were transformed to Escherichia coli BL-21 cells and seeded on Luria-Bertani medium added with 50 *μ*g/ml kanamycin (Solarbio, China) at 37°C. When the OD_600_ reached approximately 0.6, 0.5 mM isopropyl-*β*-D-thiogalactopyranoside (Solarbio, China) was added to induce the protein overexpression and then shaken-incubated at 22°C for 18 h. After harvesting by centrifugation at 4°C, 5500 rpm for 10 min, cells were resuspended in lysis buffer (500 mM NaCl, 50 mM HEPES, 5 mM imidazole, and 5% [*v*/*v*] glycerol; pH = 7.5) and disrupted by an ultrasonicator for 30 min. And the lysate was centrifugated at 4°C, 15,000 rpm for 30 min. After filtering through a 0.45 *μ*m cellulose acetate syringe filter, the supernatant containing the target protein was loaded onto HisTrap™ HP Ni-NTA columns (LOT 10236775, GE, USA). The recombinant His-tagged PPAR*γ* was purified following the instructions.

### 2.13. Biolayer Interferometry (BLI) Assay

BLI is an all-optical method to characterize blinding affinity between small molecules and WT or MT forms that was carried out on an Octet RED 96 system (ForteBio, USA) [22]. In brief, 200 *μ*l sample volumes per well were added in opaque 96-well plates. His-tagged PPAR*γ* (100 *μ*g/ml) was loaded onto Ni-nitrilotriacetic acid biosensors, which were preequilibrated in kinetics buffer (1× PBS with 0.02% Tween-20) at 30°C. The biosensors were brought in the kinetics buffer to baselines for 60 s. Then, a concentration gradient of ligustrazine solution (500, 250, 125, 62.5, and 31.25 *μ*M in PBS buffer with 0.02% Tween-20 and 0.5% DMSO) was immobilized onto Ni-nitrilotriacetic acid biosensors to develop kinetics analysis. An equal amount of DMSO was added into wells as the controls. After the association (60 s) and dissociation steps (90 s), the association and dissociation curves were fitted, and data were analyzed by Octet Data Analysis software. The affinity constant (*K*_D_) was measured as the ratio between the dissociation rate constant (*K*_off_) and the association rate constant (*K*_on_); that is, *K*_D_ = *K*_off_/*K*_on_.

### 2.14. Animal Model Preparation and Group Assignment

A total of thirty-six adult male SD rats (weighing 200 ± 20 g) were provided from the Qinglongshan Experimental Animal Breeding Farm (Nanjing, China). Rats were randomly divided into six groups (*n* = 6), that is, the sham, model, LN, sodium hyaluronate (SH), polylactic acid (PLA), and ligustrazine (LZ) groups. They were housed under standard conditions of controlled temperature (22 ± 2°C) with a reverse 12/12 h light/dark cycle (lights off at 06:00 AM) and had free access to tap water and food. The experiments were approved by the Laboratory Animal Management Committee of Nanjing University of Chinese Medicine (No. ACU171112).

The model preparation was established by previous studies [23, 24]. Briefly, rats were stopped feeding for about 12 h. After anesthesia with 1~1.5% isoflurane, a 1.5~2 cm midline incision was made after shaving and disinfection. The cecum was placed on wet gauze and scraped by dry gauze until serosal petechiae appeared on the intestinal surfaces. After exposure to air for 5 min, the cecum was replaced into the abdominal cavity and the abdominal wall was sutured. The sham group was only given laparotomy.

LN was prepared by our team as previously described [25]. A proper amount of ligustrazine was dissolved in 0.25% poloxamer solution to prepare 1 mg/ml ligustrazine solution, that is, LZ. PLA nanoparticles were prepared using the same preparation methods of LN. In the LN, SH, PLA, and LZ groups, 5 ml/kg LN, 0.5 ml/kg SH, 0.5 ml/kg PLA, and 1 mg/ml LZ were applied to the abraded peritoneum and its surrounding areas before closing the peritoneal cavity, respectively. The animal grouping and treatment are listed in Table 1. On the 7^th^ day after the operation, rats were anesthetized with 1~1.5% isoflurane. And an inverted U-shaped incision was used to open the abdominal cavity. The cecum including adhesive sites was collected for further study. After hemostasis was done completely, the abdominal wall was closed. All rats were sacrificed with cervical dislocation.

### 2.15. Macroscopic Evaluation

The adhesions were blindly evaluated and scored by two authors, individually. The adhesion scoring system was based on a five-stage adhesion score [26, 27], as shown in Table 2.

### 2.16. Histopathological Examination

A portion of the cecum tissues was dissected and fixed in 4% formaldehyde for 24 h. The tissues were dehydrated, embedded, and cut into sections using a routine method. The sections were then used for hematoxylin-eosin (HE) and Masson staining. Images were visualized under a microscope (Leica DM2500, Germany). The represented graphs were presented.

### 2.17. Immunohistochemical Assay

The cecum tissues were fixed in 4% formaldehyde for 24 h, following a series of normal procedures of deparaffinization with xylene and dehydration with alcohol step by step. And then they were embedded in paraffin wax and cut into 4 *μ*m thickness sections. These sections were immunostained with primary antibodies against CTGF, VCAM-1, and MMP2 overnight at 4°C. The second antibody and color were all conducted according to the diaminobenzidine tetrahydrochloride (DAB) kit's instructions. Views were randomly visualized under a microscope (Leica DM2500, Germany).

### 2.18. Immunofluorescence Assay

The tissue slides were baked at 65°C for 1 h, blocked in 1% bovine serum albumin, and incubated with the primary antibodies against PPAR*γ* and HIF-1*α* overnight at 4°C. After three washes with PBS, these slides were incubated with fluorescent secondary antibody for 30 min. For RPMCs or HPMCs, the cells were treated with vehicle or ligustrazine at different concentrations. After incubation with primary and second antibodies, the nuclei of cells were stained with DAPI for 5 min. The images of sections and cells were blindly observed with a microscope (Leica DMi8, Germany).

### 2.19. qRT-PCR Assay

Total RNA from PMCs or cecum tissues was prepared with TRIzol reagent (Invitrogen, USA) and used to synthesize the first-strand cDNA with Hifair®Ш 1st Strand cDNA Synthesis Kit (11141ES60, Yeasen, China). The qRT-PCR was conducted using Hieff® qPCR SYBR® Green Master Mix (High Rox) (11203ES08, Yeasen, China), and glyceraldehyde 3-phosphate dehydrogenase (GAPDH) was used as a control. The primer sequences were presented in Supplementary Table S2.

### 2.20. Western Blot Analysis

Total crude proteins were prepared from PMCs or cecum tissues by using routine protocols. Briefly, 15 *μ*l loading buffer of each group was added into sodium dodecyl sulfate-polyacrylamide gel electrophoresis. The separated proteins were transferred onto PVDF membranes, which were then incubated with primary antibodies overnight at 4°C. After three washes with TBST, the membranes were incubated with secondary antibodies at room temperature for 80 min. Finally, the band visualization was viewed by the Chemiluminescence Imaging System (Bio-Rad, USA).

### 2.21. Bioinformatics Analysis

The mRNA microarray expression profile datasets were retrieved and downloaded from the GEO database (available online: http://www.ncbi.nlm.nih.gov/geo) by searching the following keywords: “RNA”, “peritoneal adhesion” or “abdominal adhesion”, and “Mus musculus” (organism). After screening, one mRNA expression dataset GSE4715 was selected for analysis. The data were downloaded and subjected to Morpheus online tool (https://software.broadinstitute.org/morpheus/). The significantly expressed genes were identified when the signal to noise > 1 or signal to noise < −1. GO annotation and KEGG enrichment were conducted by the Database for Annotation, Visualization, and Integrated Discovery (DAVID) [29] when *P* value < 0.05 was considered as a screening threshold.

### 2.22. Statistics

All results were expressed as the mean ± standard deviation. The data among multigroups were analyzed by one-way analysis of variance (ANOVA) with LSD test, and the data between two groups were analyzed using *t*-test. The Kruskal-Wallis test was used for nonnormally distributed continuous data. *P* < 0.05 was considered significantly different.

## 3. Results

### 3.1. Ligustrazine Suppresses the Production of Profibrotic Cytokines in Hypoxia-Induced PMCs

**Figure 1:**
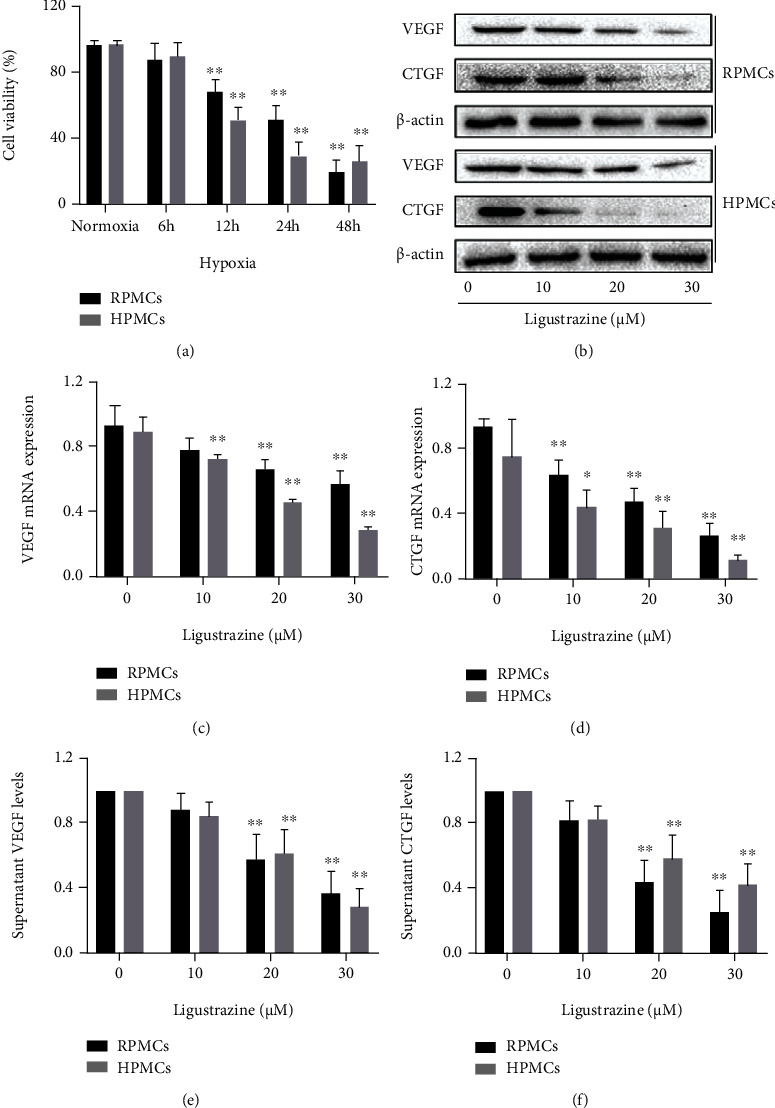
Ligustrazine suppresses the production of profibrotic cytokines in hypoxia-induced PMCs. (a) MTT assay of PMCs under hypoxic conditions with different exposure time. Compared with the control, ^∗^*P* < 0.05 and ^∗∗^*P* < 0.01. (b) Western blot analysis of protein expression of profibrotic cytokines (VEGF and CTGF) in hypoxia-induced PMCs treated with different concentrations of ligustrazine for 24 h. (c, d) qRT-PCR analysis of mRNA levels of profibrotic cytokines (VEGF and CTGF) in hypoxia-induced PMCs treated with different concentrations of ligustrazine for 24 h. Compared with the control, ^∗^*P* < 0.05 and ^∗∗^*P* < 0.01. (e, f) ELISA analysis of supernatant levels of profibrotic cytokines (VEGF and CTGF) in hypoxia-induced PMCs treated with different concentrations of ligustrazine for 24 h. Compared with the control, ^∗^*P* < 0.05 and ^∗∗^*P* < 0.01.

PMCs were cultured under hypoxic conditions at four different time (6, 12, 24, and 48 h), respectively. It showed that cell viability was decreased depending on the different exposure time (Figure 1(a)). Because hypoxic conditions for 24 h could reduce the viability of 50% PMCs, this exposure time was used in the subsequent study. VEGF and CTGF are the two pivotal profibrotic cytokines in PMCs. We preliminarily found that ligustrazine reduced the protein expression and mRNA levels of the two profibrotic cytokines in a concentration-dependent manner (Figures 1(b)–1(d)). And the supernatant levels of VEGF and CTGF were concentration-dependently decreased (Figures 1(e) and 1(f)). Taken together, the results suggested that ligustrazine could suppress the production of profibrotic cytokines in hypoxia-induced PMCs.

### 3.2. Ligustrazine Inhibits the Expression of Migration and Adhesion-Associated Molecules in Hypoxia-Induced PMCs

**Figure 2:**
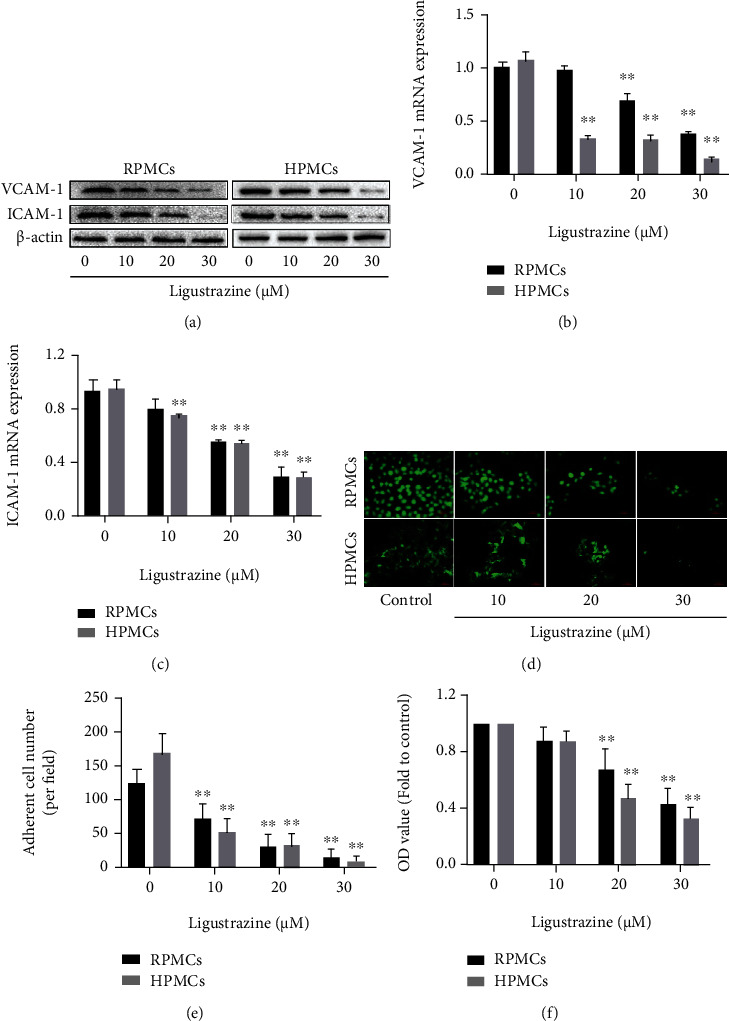
Ligustrazine inhibits the expression of migration and adhesion-associated molecules in hypoxia-induced PMCs. (a) Western blot analysis of protein expression of migration and adhesion-associated molecules (VCAM-1 and ICAM-1) in hypoxia-induced PMCs treated with different concentrations of ligustrazine for 24 h. (b, c) qRT-PCR analysis of mRNA levels of migration and adhesion-associated molecules (VCAM-1 and ICAM-1) in hypoxia-induced PMCs treated with different concentrations of ligustrazine for 24 h. Compared with the control, ^∗^*P* < 0.05 and ^∗∗^*P* < 0.01. (d, e) FDA staining assay of cell viability in hypoxia-induced PMCs treated with different concentrations of ligustrazine for 24 h (200x). Compared with the control, ^∗^*P* < 0.05 and ^∗∗^*P* < 0.01. (f) MTT analysis of OD values in hypoxia-induced PMCs treated with different concentrations of ligustrazine for 24 h. Compared with the control, ^∗^*P* < 0.05 and ^∗∗^*P* < 0.01.

Cell migration and adhesion are key steps towards peritoneal adhesion, during which many adhesion molecules are involved in. VCAM-1 and ICAM-1 are identified as two important biomarkers in cell migration and adhesion [6]. The protein expression of both adhesion contributors was decreased by ligustrazine in a concentration-dependent manner (Figure 2(a)). The results were consistent with those of qRT-PCR analysis. The mRNA levels of VCAM-1 and ICAM-1 were downregulated with the increased concentration of ligustrazine (Figures 2(b) and 2(c)). Moreover, FDA morphology staining (Figures 2(d) and 2(e)) and MTT analysis (Figure 2(f)) indicated that the viability of hypoxia-induced PMCs was restricted by ligustrazine. Together, the discoveries indicated that ligustrazine could inhibit the expression of migration and adhesion-associated molecules in hypoxia-induced PMCs.

### 3.3. Ligustrazine Represses the Expression of Cytoskeleton Proteins in Hypoxia-Induced PMCs

**Figure 3:**
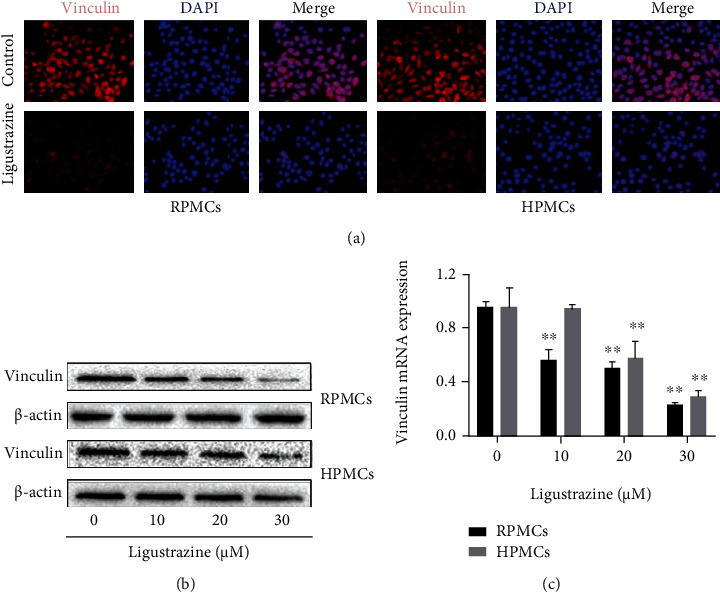
Ligustrazine represses the expression of cytoskeleton proteins in hypoxia-induced PMCs. (a) Immunofluorescence analysis of cytoskeleton protein (vinculin) expression in hypoxia-induced PMCs treated with 20 *μ*M ligustrazine for 24 h (200x). (b) Western blot analysis of cytoskeleton protein (vinculin) expression in hypoxia-induced PMCs treated with different concentrations of ligustrazine for 24 h. (c) qRT-PCR analysis of mRNA levels of cytoskeleton molecules (vinculin) in hypoxia-induced PMCs treated with different concentrations of ligustrazine for 24 h. Compared with the control, ^∗^*P* < 0.05 and ^∗∗^*P* < 0.01.

Cytoskeleton acts a meaningful role in cellular function, and it is closely related to the activation of the fibrinolysis system in PMCs [30]. To determine the exact roles of ligustrazine on cytoskeletal change of PMCs under hypoxia, the cytoskeleton protein expression of vinculin was measured at a concentrated point. We found that ligustrazine could significantly reduce the expression of vinculin, evidenced by immunofluorescence analysis (Figure 3(a)). It also demonstrated that ligustrazine concentration-dependently decreased the expression of vinculin, evidenced by Western blot and qRT-PCR analysis (Figures 3(b) and 3(c)). Taken together, the results revealed that ligustrazine could repress the expression of cytoskeleton proteins in hypoxia-induced PMCs.

### 3.4. Ligustrazine Restricts Hypoxia-Induced PMCs to Obtain Myofibroblast-Like Phenotypes

**Figure 4:**
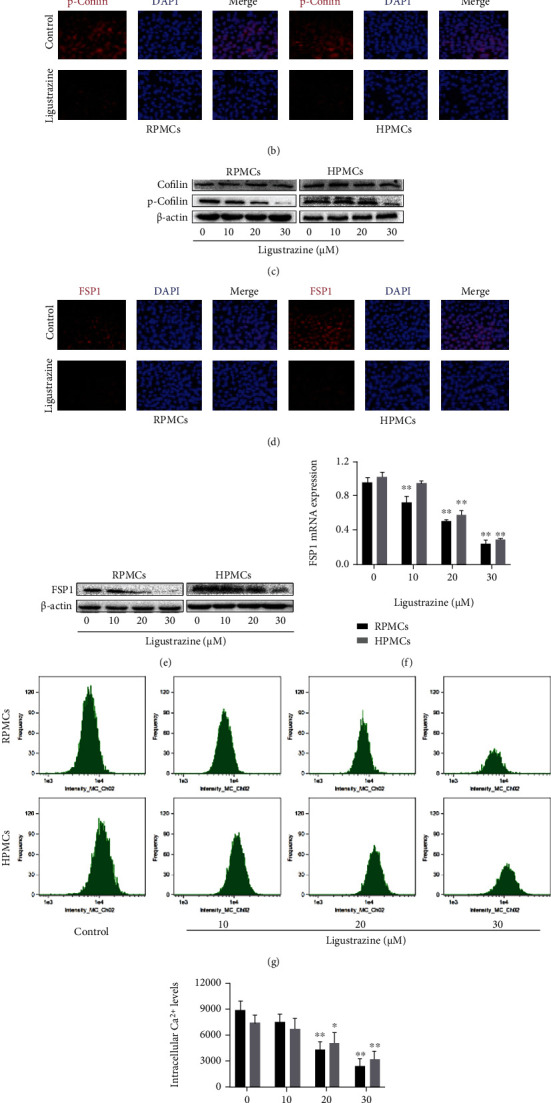
Ligustrazine restricts hypoxia-induced PMCs to obtain myofibroblast-like phenotypes. (a) F-Actin staining assay to assess cytoskeletal change in hypoxia-induced PMCs treated with 20 *μ*M ligustrazine for 24 h (200x). (b) Immunofluorescence assay of p-cofilin in hypoxia-induced PMCs treated with 20 *μ*M ligustrazine for 24 h (200x). (c) Western blot analysis of cofilin and p-cofilin expression in hypoxia-induced PMCs treated with different concentrations of ligustrazine for 24 h. (d) Immunofluorescence assay of FSP1 in hypoxia-induced PMCs treated with 20 *μ*M ligustrazine for 24 h (200x). (e, f) Western blot and qRT-PCR analysis of FSP1 levels in hypoxia-induced PMCs treated with different concentrations of ligustrazine for 24 h. Compared with the control, ^∗^*P* < 0.05 and ^∗∗^*P* < 0.01. (g, h) Flow cytometry analysis of intracellular Ca^2+^ levels in hypoxia-induced PMCs treated with different concentrations of ligustrazine for 24 h. Compared with the control, ^∗^*P* < 0.05 and ^∗∗^*P* < 0.01.

Cytoskeletal change might give us important information about the critical function of cells [31]. And the reorganization of F-actin microfilament plays an important role in the cytoskeleton. Herein, the cytoskeleton was stained with F-actin using phalloidin, which is represented by a green color in Figure 4(a). It demonstrated that ligustrazine could significantly reduce the formation of actin stress fibers. Considering the central roles of cofilin in cytoskeletal stability [32], we also detected the levels of the biomarker cofilin and its phosphorylation status under hypoxic conditions, which indicated that p-cofilin was significantly decreased by ligustrazine, evidenced by immunofluorescence assay (Figure 4(b)). The results were also in line with those of Western blot analysis. It indicated that the protein expression of p-cofilin was decreased by ligustrazine in a concentration-dependent manner (Figure 4(c)). It is reported that PMCs after the peritoneal injury can transform into myofibroblasts, which is the key step of the developmental progress of peritoneal adhesion or fibrosis [33, 34]. FSP1 acts as an important contributor to fibroblast and myofibroblast [35]. The immunofluorescence results demonstrated that ligustrazine could markedly decrease the expression of FSP1 (Figure 4(d)). Western blot and qRT-PCR analysis showed that the protein expression and mRNA levels of FSP1 were downregulated in a concentration-dependent manner (Figures 4(e) and 4(f)). Besides, intracellular Ca^2+^ levels were concentration-dependently decreased, as presented by flow cytometry assay (Figures 4(g) and 4(h)). Collectively, the findings revealed that ligustrazine could restrict hypoxia-induced PMCs to obtain myofibroblast-like phenotypes.

### 3.5. Ligustrazine Inhibits Hypoxia-Induced PMC ECM Deposition and EMT Transition

**Figure 5:**
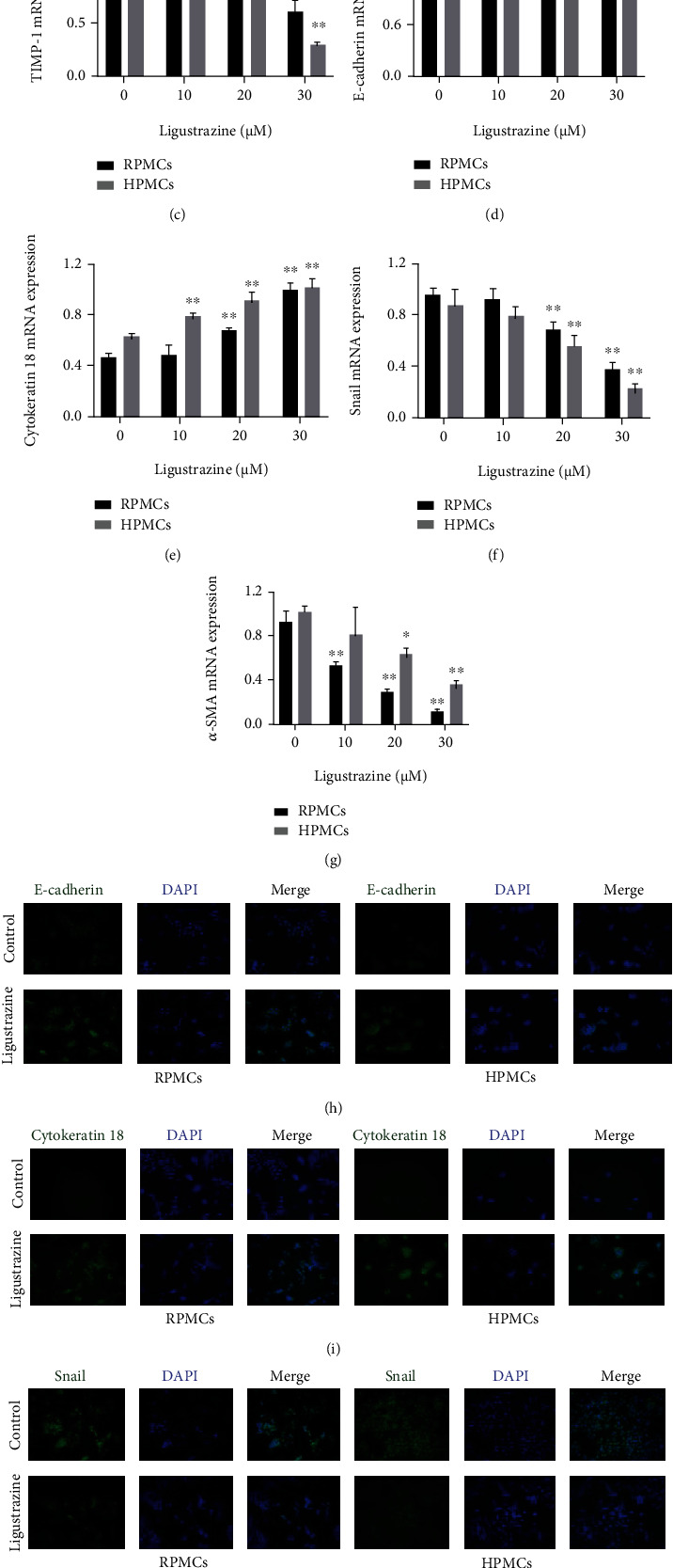
Ligustrazine inhibits hypoxia-induced PMC ECM deposition and EMT transition. (a) Western blot analysis of protein expression of ECM-associated molecules (MMP2 and TIMP-1), mesothelial-related phenotypic biomarkers (E-cadherin and cytokeratin 18), and mesenchymal-related phenotypic biomarkers (Snail and *α*-SMA) in hypoxia-induced PMCs treated with different concentrations of ligustrazine for 24 h. (b–g) qRT-PCR analysis of mRNA levels of ECM-associated molecules (MMP2 and TIMP-1), mesothelial-related phenotypic biomarkers (E-cadherin and cytokeratin 18), and mesenchymal-related phenotypic biomarkers (Snail and *α*-SMA) in hypoxia-induced PMCs treated with different concentrations of ligustrazine for 24 h. Compared with the control, ^∗^*P* < 0.05 and ^∗∗^*P* < 0.01. (h–k) Immunofluorescence assay of mesothelial-related phenotypic biomarkers (E-cadherin, Cytokeratin 18), and mesenchymal-related phenotypic biomarkers (Snail, *α*-SMA) in hypoxia-induced PMCs treated with 20 *μ*M ligustrazine for 24 h (200x).

ECM remodeling and EMT transition are critical steps toward the accumulation of myofibroblasts in the pathological process of peritoneal fibrosis, during which many genetic transcripts are involved [33]. ECM-associated molecules (MMP2 and TIMP-1) were measured at a concentrated point. It suggested that protein expression and mRNA levels of MMP2 and TIMP-1 were concentration-dependently downexpressed by ligustrazine (Figures 5(a)–5(c)). Meanwhile, the mesothelial-related phenotypic biomarkers (E-cadherin and cytokeratin 18) and mesenchymal-related phenotypic biomarkers (Snail and *α*-SMA) were determined in hypoxia-induced PMCs treated with different concentrations of ligustrazine, respectively. Interestingly, we found that ligustrazine could increase the expression of mesothelial cell biomarkers and decrease the expression of mesenchymal cell biomarkers in a dose-dependent manner (Figures 5(a) and 5(d)–5(g)). 20 *μ*M ligustrazine could significantly upregulate the levels of E-cadherin and cytokeratin 18 and downregulate the levels of Snail and *α*-SMA in immunofluorescence analysis (Figures 5(h)–5(k)). Altogether, these data demonstrated that ligustrazine could inhibit hypoxia-induced PMC ECM remodeling and EMT transition.

### 3.6. Ligustrazine Suppresses Hypoxia-Induced PMC Functions by Activating PPAR*γ*

**Figure 6:**
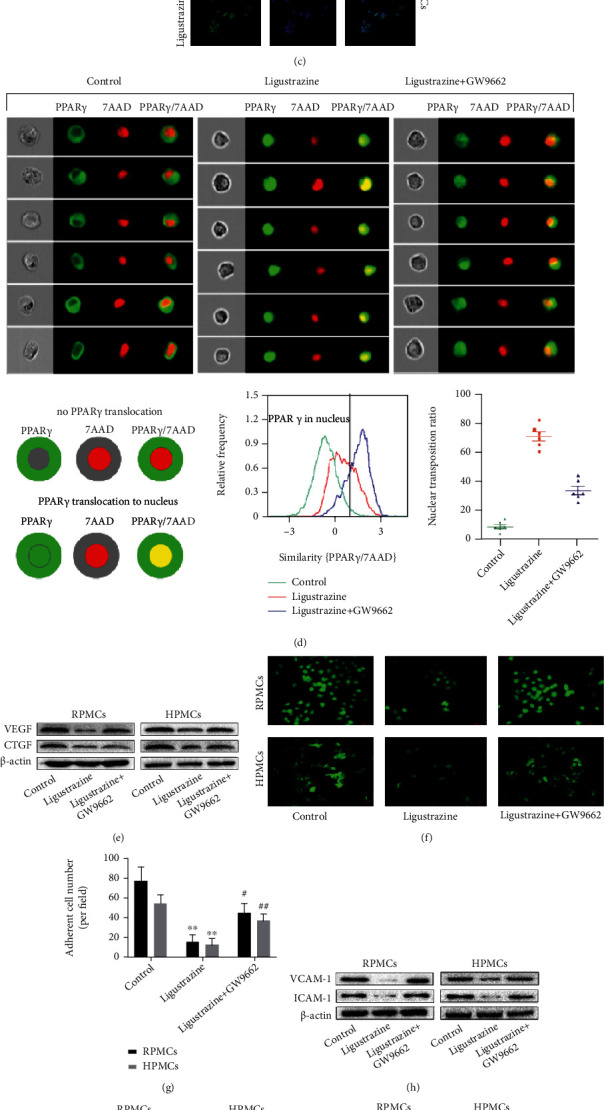
Ligustrazine suppresses hypoxia-induced PMC functions by activating PPAR*γ*. (a, b) Western blot and qRT-PCR analysis of PPAR*γ* levels in hypoxia-induced PMCs treated with different concentrations of ligustrazine for 24 h. Compared with the control, ^∗^*P* < 0.05 and ^∗∗^*P* < 0.01. (c) Immunofluorescence analysis of PPAR*γ* nuclear distribution in hypoxia-induced PMCs treated with 20 *μ*M ligustrazine for 24 h (200x). (d) Flow cytometry analysis of PPAR*γ* nuclear translocation in hypoxia-induced PMCs treated with 20 *μ*M ligustrazine and/or 1 *μ*M GW9662 for 24 h. (e) Western blot analysis of protein expression of profibrotic cytokines (VEGF and CTGF) in hypoxia-induced PMCs treated with 20 *μ*M ligustrazine and/or 1 *μ*M GW9662 for 24 h. (f, g) FDA staining assay of cell viability in hypoxia-induced PMCs treated with 20 *μ*M ligustrazine and/or 1 *μ*M GW9662 for 24 h (200x). Compared with the control, ^∗^*P* < 0.05 and ^∗∗^*P* < 0.01. Compared with the ligustrazine group, ^#^*P* < 0.05 and ^##^*P* < 0.01. (h) Western blot analysis of protein expression of migration and adhesion-associated molecules (VCAM-1 and ICAM-1) in hypoxia-induced PMCs treated with 20 *μ*M ligustrazine and/or 1 *μ*M GW9662 for 24 h. (i) Western blot analysis of cytoskeleton protein (vinculin) expression in hypoxia-induced PMCs treated with 20 *μ*M ligustrazine and/or 1 *μ*M GW9662 for 24 h. (j) Western blot analysis of protein expression of FSP1 in hypoxia-induced PMCs treated with 20 *μ*M ligustrazine and/or 1 *μ*M GW9662 for 24 h. (k) Western blot analysis of protein expression of ECM-associated molecules (MMP2 and TIMP-1), mesothelial-related phenotypic biomarkers (E-cadherin and cytokeratin 18), and mesenchymal-related phenotypic biomarkers (Snail and *α*-SMA) in hypoxia-induced PMCs treated with 20 *μ*M ligustrazine and/or 1 *μ*M GW9662 for 24 h.

Our previous study has shown that ligustrazine has positive effects on postoperative peritoneal adhesion through activating the inflammatory signaling pathway [16]. PPAR*γ* is regarded as a target biomarker that has a direct effect on PMC biology [36]. To determine the exact role of the nuclear receptor in the biological function of ligustrazine on hypoxia-induced PMC transition, the nuclear protein expression and mRNA levels of PPAR*γ* were preliminarily measured using Western blot and qRT-PCR. It indicated that ligustrazine concentration-dependently increased the protein expression and mRNA levels of PPAR*γ* (Figures 6(a) and 6(b)), which was in line with the results of immunofluorescence analysis (Figure 6(c)). GW9662 as an antagonist of PPAR*γ* [37] was used to further verify the critical function of PPAR*γ*. The results of flow cytometry analysis showed that the ligustrazine-driven nuclear distribution of PPAR*γ* was reversed by GW9662 (Figure 6(d)). We found that GW9662 reversed the suppression effect of ligustrazine on the production of profibrotic cytokines (VEGF and CTGF) (Figure 6(e)). And GW9662 restored the inhibitory effect of ligustrazine on the expression of migration and adhesion-associated molecules (VCAM-1 and ICAM-1) (Figures 6(f)–6(h)). GW9662 also reversed the restriction effect of ligustrazine on the expression of cytoskeleton proteins (vinculin) (Figure 6(i)) and FSP1 (Figure 6(j)). Besides, GW9662 relieved the suppression effect of ligustrazine on hypoxia-induced PMC ECM accumulation and EMT changes (Figure 6(k)). Collectively, the results demonstrated that the suppression effect of ligustrazine on hypoxia-induced PMC functions was achieved by regulating PPAR*γ*.

### 3.7. The Suppression Effect of Ligustrazine Is Achieved by the Activated PPAR*γ* on the Transrepression of SMRT-Mediated HIF-1*α*

**Figure 7:**
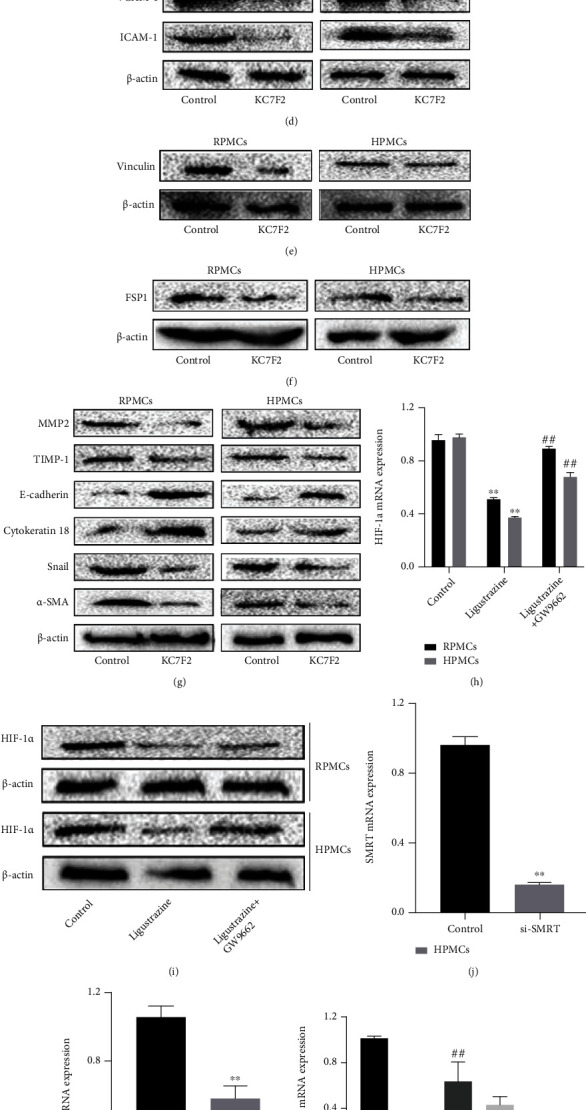
The suppression effect of ligustrazine is achieved by the activated PPAR*γ* on the transrepression of SMRT-mediated HIF-1*α*. (a) Western blot analysis of protein expression of profibrotic cytokines (VEGF and CTGF) in hypoxia-induced PMCs treated with KC7F2 for 24 h. (b, c) FDA staining assay of cell viability in hypoxia-induced PMCs treated with KC7F2 for 24 h (200x). Compared with the control, ^∗^*P* < 0.05 and ^∗∗^*P* < 0.01. (d) Western blot analysis of protein expression of migration and adhesion-associated molecules (VCAM-1 and ICAM-1) in hypoxia-induced PMCs treated with KC7F2 for 24 h. (e) Western blot analysis of cytoskeleton protein (vinculin) expression in hypoxia-induced PMCs treated with KC7F2 for 24 h. (f) Western blot analysis of protein expression of FSP1 in hypoxia-induced PMCs treated with KC7F2 for 24 h. (g) Western blot analysis of protein expression of ECM-associated molecules (MMP2 and TIMP-1), mesothelial-related phenotypic biomarkers (E-cadherin and cytokeratin 18), and mesenchymal-related phenotypic biomarkers (Snail and *α*-SMA) in hypoxia-induced PMCs treated with KC7F2 for 24 h. (h, i) qRT-PCR and Western blot analysis of HIF-1*α* levels in hypoxia-induced PMCs treated with 20 *μ*M ligustrazine and/or 1 *μ*M GW9662 for 24 h. Compared with the control, ^∗^*P* < 0.05 and ^∗∗^*P* < 0.01. Compared with the ligustrazine group, ^#^*P* < 0.05 and ^##^*P* < 0.01. (j, k) qRT-PCR analysis of mRNA levels of SMRT and NCoR in hypoxia-induced HPMCs after different transfection for 24 h. Compared with the control, ^∗^*P* < 0.05 and ^∗∗^*P* < 0.01. (l) qRT-PCR analysis of mRNA levels of HIF-1*α* in hypoxia-induced HPMCs treated with 20 *μ*M ligustrazine and/or with different transfection for 24 h. Compared with the control, ^∗^*P* < 0.05 and ^∗∗^*P* < 0.01. Compared with the ligustrazine group, ^#^*P* < 0.05 and ^##^*P* < 0.01. (m) Western blot analysis of protein expression of HIF-1*α* in hypoxia-induced HPMCs treated with 20 *μ*M ligustrazine and/or with different transfection for 24 h.

HIF-1*α* induced by hypoxic conditions is a critical transcription factor involved in the pathophysiologic process of peritoneal injury [38]. To this point, the HIF-1*α* inhibitor KC7F2 (50 *μ*M) was applied to determine the transcriptional role of HIF-1*α* in the EMT process of PMCs. It indicated that KC7F2 downregulated the production of the profibrotic cytokines, restricted the expression of migration and adhesion-associated molecules, and decreased the expression of cytoskeleton protein (Figures 7(a)–7(e)). Moreover, KC7F2 suppressed FSP1 expression and inhibited ECM recruitment and EMT transition (Figures 7(f) and 7(g)). The above data indicated that the HIF-1*α* played key roles in hypoxia-induced PMC functions. The mRNA levels and protein expression of HIF-1*α* were downregulated by ligustrazine, which were reversed by GW9662 (Figures 7(h) and 7(i)). It demonstrated that the HIF-1*α* expression suppressed by ligustrazine was achieved by regulating PPAR*γ*. It reported that the transcriptional activity of PPAR*γ* was regulated by the recruitment of NCoR or SMRT [39]. In our study, we found that the mRNA levels and protein expression of HIF-1*α* inhibited by ligustrazine were dramatically reversed after transfection with si-SMRT in HPMCs (Figures 7(j)–7(m)). However, there was no change in the si-NCoR group. Taken together, it showed that the suppression effect of ligustrazine was achieved by the activated PPAR*γ* on the transrepression of SMRT-mediated HIF-1*α*.

### 3.8. Molecular Docking Assay of Ligustrazine and PPAR*γ*

**Figure 8:**
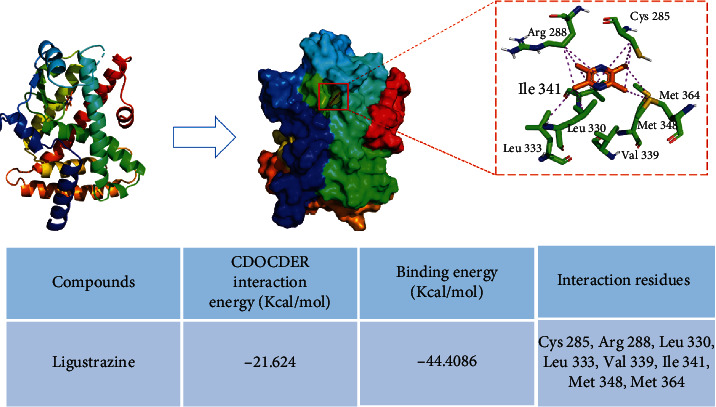
Molecular docking assay of ligustrazine and PPAR*γ*. Several potential binding pocket residues (Cys 285, Arg 288, Leu 330, Leu 333, Val 339, Ile 341, Met 348, and Met 364) were predicted.

The binding mode of ligustrazine and PPAR*γ* was predicted by using the molecular docking method (Figure 8). The binding site of the ligustrazine/PPAR*γ* complex revealed several binding pocket residues (Cys 285, Arg 288, Leu 330, Leu 333, Val 339, Ile 341, Met 348, and Met 364). All of them formed one or two alkyl interactions with methyl of ligustrazine, and each residue interacted with at least one of the four methyls (no more than 2 methyls). Besides, Cys 285, Arg 288, and Ile 341 formed additional Pi-Alkyl interactions with the pyrazine ring, which clearly emphasized the importance of hydrophobic bonds in ligustrazine. Altogether, these results suggested that there were binding sites between ligustrazine and PPAR*γ*, thereby leading to a series of subsequent biological processes.

### 3.9. Ligustrazine Bound Directly to PPAR*γ*, and Val 339/Ile 341 Residue Was Critical for the Binding of PPAR*γ* to Ligustrazine

**Figure 9:**
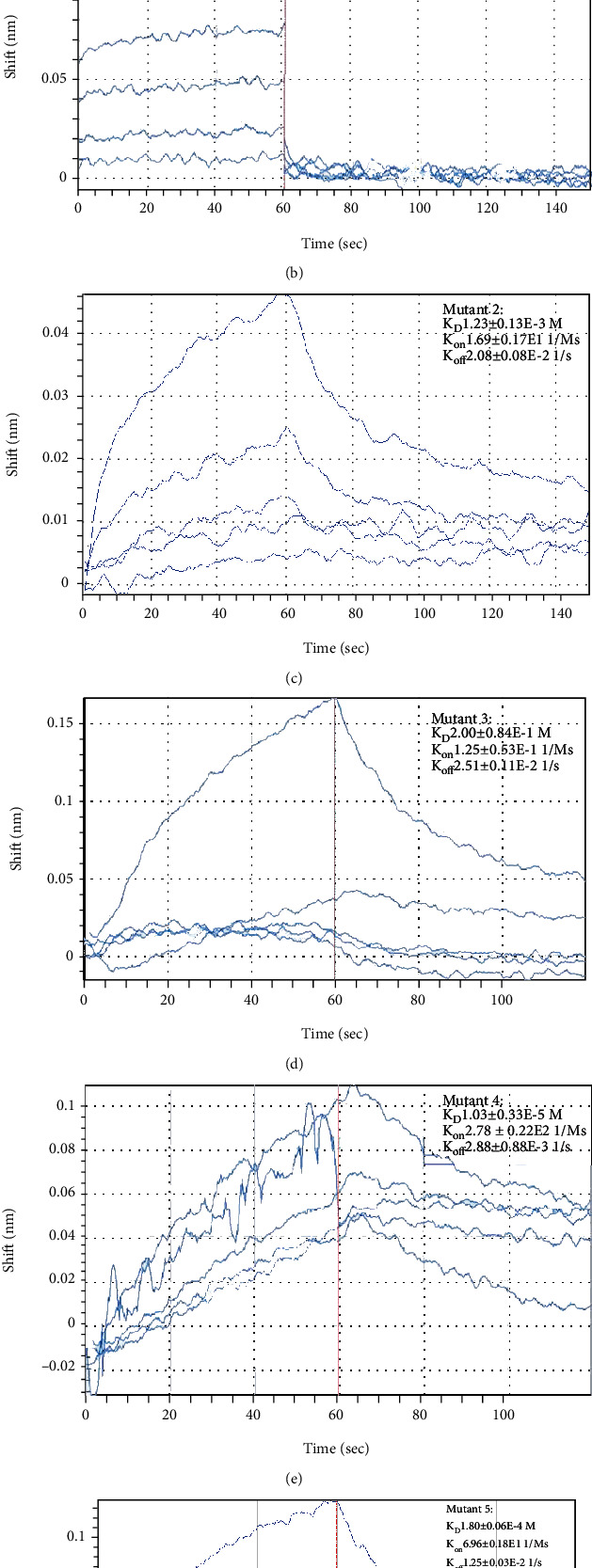
Val 339/Ile 341 residue was critical for the binding of PPAR*γ* to ligustrazine. (a) The binding of ligustrazine to PPAR*γ* (WT) was conducted by the BLI assay. (b) The binding of mutant 1 was conducted by the BLI assay. (c) The binding of mutant 2 was conducted by the BLI assay. (d) The binding of mutant 3 was conducted by the BLI assay. (e) The binding of mutant 4 was conducted by the BLI assay. (f) The binding of mutant 5 was conducted by the BLI assay.

The affinity binding between ligustrazine and PPAR*γ* is a critical step to initiate signaling transduction. To further ensure the specific binding effects, the BLI assay was used to calculate the rates of biomolecular kinetics. The affinity results between ligustrazine and WT PPAR*γ* indicated that ligustrazine had an affinity function for PPAR*γ* of 3.94 ± 0.27*E* − 5 M. The *K*_on_ and *K*_off_ values of ligustrazine for WT PPAR*γ* were 1.59 ± 0.09*E*3 1/Ms and 6.25 ± 0.22*E* − 2 1/s, respectively (Figure 9(a)). And the affinities of mutant forms of PPAR*γ* to ligustrazine showed that the *K*_D_ values of mutant 1, mutant 2, mutant 3, mutant 4, and mutant 5 were 2.37 ± 0.51*E* − 3 M, 1.23 ± 0.13*E* − 3 M, 2.00 ± 0.84*E* − 1 M, 1.03 ± 0.33*E* − 5 M, and 1.80 ± 0.06*E* − 4 M, respectively (Figures 9(b)–9(f)). The affinity of WT PPAR*γ* to ligustrazine was about 60, 31, 4953, 0.3, and 4.6 times higher than that of mutant 1, mutant 2, mutant 3, mutant 4, and mutant 5. The binding strength between ligustrazine and PPAR*γ* could be listed as follows: mutant 4 > mutant 5 > mutant 2 > mutant 1 > mutant 3. All discoveries revealed that ligustrazine is directly bound to PPAR*γ*, and mutant 3 displayed the weakest interaction with ligustrazine than the other four mutants, suggesting the Val 339/Ile 341 residue was critical for the binding of PPAR*γ* to ligustrazine.

### 3.10. PPAR*γ* Pathway Identification by Bioinformatics Analysis

**Figure 10:**
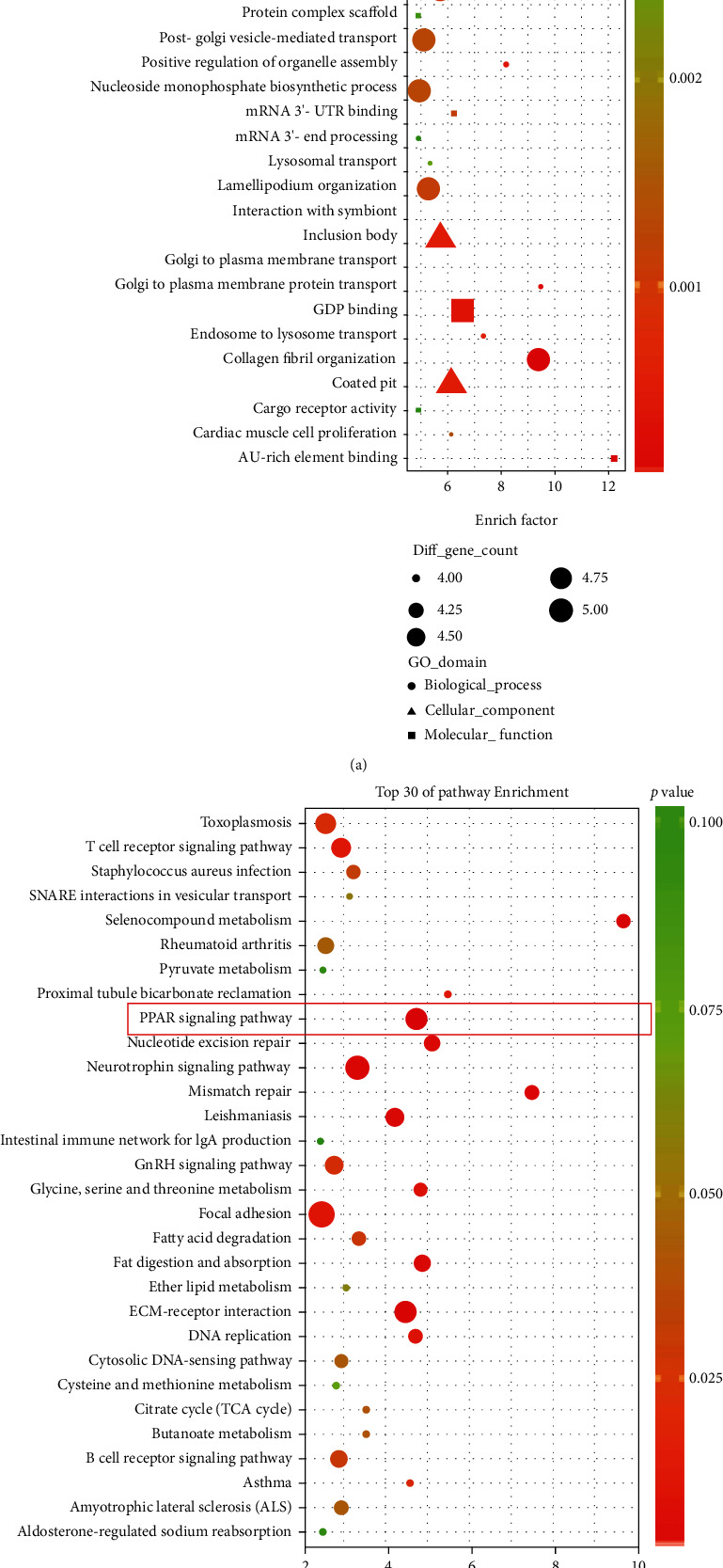
GO annotation and pathway enrichment of the significantly expressed genes associated with PPA. (a) GO annotation results of the significantly expressed genes (top 30). (b) Pathway enrichment results of the significantly expressed genes (top 30).

The GSE4715 expression profile dataset consists of 6144 gene probes. A total of 274 differently expressed genes were identified, among which 115 were upregulated and 159 were downregulated. The 274 genes were then conducted using DAVID for biological process annotation and KEGG pathway enrichment. The GO term assignment analysis indicated that the enriched GOs in the biological process included retinoic acid receptor signaling pathway (GO: 0048384), Golgi to plasma membrane protein transport (GO: 0043001), collagen fibril organization (GO: 0030199), and serine family amino acid metabolic process (GO: 0009069), as viewed in Figure 10(a). Based on the pathway enrichment analysis, the altered genes were involved in the PPAR signaling pathway (top 1) and ECM-receptor interaction (Figure 10(b)). These findings further verified the critical roles of the PPAR pathway in the biological process of PPA.

### 3.11. LN Attenuates Peritoneal Adhesion in PPA Rats by Inhibiting PMC Functions

**Figure 11:**
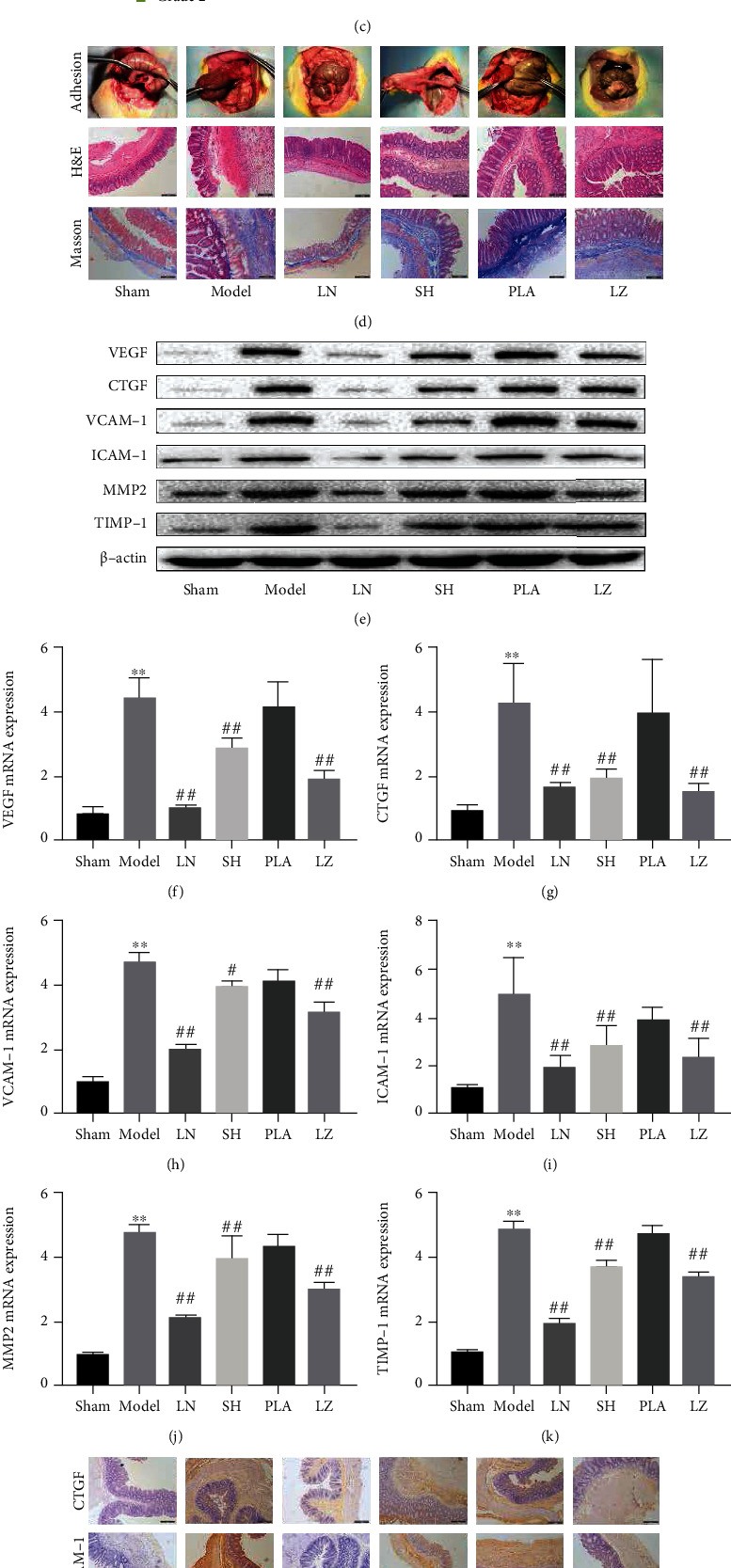
LN attenuates peritoneal adhesion in PPA rats by inhibiting PMC functions. (a) Scanning electron microscope image of LN (×200 nm). (b, c) Adhesion scores and frequency of different groups. Compared with the sham group, ^∗^*P* < 0.05 and ^∗∗^*P* < 0.01. Compared with the model group, ^#^*P* < 0.05 and ^##^*P* < 0.01. (d) Representative images of adhesion formation, HE, and Masson staining of cecum sections in different groups (100x). (e) Western blot analysis of protein expression of profibrotic cytokines (VEGF and CTGF), migration and adhesion-associated molecules (VCAM-1 and ICAM-1), and ECM-associated molecules (MMP2 and TIMP-1) in different groups. (f–k) qRT-PCR analysis of mRNA levels of profibrotic cytokines (VEGF and CTGF), migration and adhesion-associated molecules (VCAM-1 and ICAM-1), and ECM-associated molecules (MMP2 and TIMP-1) in different groups. Compared with the sham group, ^∗^*P* < 0.05 and ^∗∗^*P* < 0.01. Compared with the model group, ^#^*P* < 0.05 and ^##^*P* < 0.01. (l) Immunohistochemistry analysis of profibrotic cytokine (CTGF), migration and adhesion-associated molecule (VCAM-1), and ECM-associated molecule (MMP2) in different groups (100x). (m) Immunofluorescence analysis of PPAR*γ* and HIF-1*α* in different groups (100x).

Loading capacity and entrapment efficiency are two pivotal indicators to measure the properties of nanoparticles. The PLA nanoparticles loaded with ligustrazine had high loading capacity and entrapment efficiency, as we reported previously [25]. It also had uniform spherical morphology with a smooth surface and good dispersivity (Figure 11(a)). The distribution of particle size is about 200 nm. And LN had stable and sustained release behavior, evidenced by the in vitro drug release assay [25]. The outstanding features of LN are the prominently sustained release effect and good stability for tissue penetration via a positive targeting mechanism, which may be a novel agent for preventing PPA. We found that the incision of all rats was primary healing, without infection or other complications during the model preparation. No rat death was observed, and there was no significant difference in body weight among the six groups. Two rats in the model group had dark black cecum, which might have intestinal obstruction or necrosis. One rat in the PLA group had swelling cecum, which was smelly after incision. All rats were sacrificed on the 7^th^ day after successfully modeling. The adhesion scores and frequency of different grades among groups are displayed in Figures 11(b) and 11(c). The adhesion scores were listed as follows: model > PLA > LZ > SH> LN > Sham. Compared with the sham group, the model group showed a severe peritoneal adhesion with a significantly higher adhesion score. In comparison with the model group, the LN group had a markedly lower adhesion score. The representative images of peritoneal adhesion in six groups are presented in Figure 11(d). Masson staining analysis showed that massive inflammatory cells and collagen fibers were found in the model group compared with the sham group. The LN group displayed a decreased fibrin thickness in comparison with the model group, which revealed fewer collagen fiber depositions (Figure 11(d)). The protein expression and mRNA levels of the profibrotic cytokines (VEGF and CTGF), migration and adhesion-associated molecules (VCAM-1 and ICAM-1), and ECM-associated molecules (MMP2 and TIMP-1) were elevated in the model group. After LN treatment, they were all significantly downregulated in the LN group (Figures 11(e)–11(k)). The results were in line with those of immunohistochemical analysis (Figure 11(l)). Additionally, we found that the expression of PPAR*γ* was increased and HIF-1*α* was downregulated after LN treatment, as evidenced by the immunofluorescence results (Figure 11(m)). Together, these findings demonstrated that LN significantly attenuated peritoneal adhesion by suppressing the production of profibrotic cytokines, inhibiting the expression of migration and adhesion-associated molecules, ECM deposition, and EMT transition, which were in line with the results of cell experiments.

## 4. Discussion

In this study, we initially demonstrated the specific roles of ligustrazine in preventing PPA at molecular levels. Our findings indicated that ligustrazine could significantly reverse hypoxia-induced PMC EMT-like changes on the cellular levels and in the rodent models. The benefit of ligustrazine on ECM remodeling and EMT transition is contributed to the activation of PPAR*γ* on transrepression of HIF-1*α* in an SMRT-dependent manner. Molecular docking and site-directed mutagenesis tests further verified the close interaction of ligustrazine with PPAR*γ*. Additionally, ligustrazine could suppress the production of profibrotic cytokines, inhibit the expression of migration and adhesion-associated molecules, and restrict the expression of cytoskeleton proteins. Herein, ligustrazine could be served as a potential strategy for the prevention of PPA.

Although surgical procedures, especially laparoscopic surgery, have made considerable progress, PPA remains a concerning problem. After the peritoneal injury, a series of cascade reactions are triggered, during which hypoxia plays the central role. Mounting evidence suggests the close relations between hypoxia and adhesion. Hypoxia remarkably alters various biomarker generations, irreversibly induces adhesion phenotype and inflammatory phenotype development [40, 41], and simultaneously increases the deposition of extracellular matrix [42], following enhancing EMT process [38] in vitro studies. To induce hypoxic conditions, PMCs were cultured under 1% O_2_, 94% N_2_, and 5% CO_2_ at different time. The cell viability of PMCs was in a time-dependent manner, which indicated that hypoxia was indeed involved in the pathogenesis of adhesion formation. The pharmacology study showed that ligustrazine had special effects on anti-inflammatory activities [43]. Our previous studies demonstrated that ligustrazine has positive preventive effects on PPA both *in vivo* and *in vitro* [16, 17]. Herein, we sought to clarify the functional roles of ligustrazine in the key pathological link of the occurrence and development of peritoneal adhesion formation. Peritoneal fibrosis is one of the major factors of PPA formation. We found that ligustrazine could downregulate the expression of profibrotic cytokines and abolish the expression of migration and adhesion-associated molecules. The result was consistent with a previous study. It indicated that ligustrazine had positive effects on antifibrosis and prevented PMCs from injury [17]. Cytoskeletal change is a prerequisite for adhesion formation, which is necessary for cell migration [31]. In the current study, we found that ligustrazine could restrict PMC phenotypic transition to acquire myofibroblast-like phenotypes, and inhibit cytoskeletal remodeling reorganization. Hypoxia can induce adhesive phenotype to peritoneal fibroblast or myofibroblast change and promote ECM accumulation and EMT transition during the following progress [31]. Moreover, the altered expression of EMT phenotypic markers contributes to the increased acquisition of myofibroblast-like phenotype and is critical to ECM remodeling. Ligustrazine was found to suppress hypoxia-induced PMC ECM deposition and EMT transition effectively, but the underlying functional mechanisms needed to be further elucidated.

In this study, we highlighted PPAR*γ* as a pivotal factor that is responsible for EMT-like changes and causes abnormal ECM accumulation in the effector mechanisms of ligustrazine. Mechanistically, ligustrazine activated the nuclear distribution of PPAR*γ*. The prior observations were all reversed by the knocking down of PPAR*γ*. And the results of the following bioinformatics analysis further confirmed our hypothesis that the activation of PPAR*γ* is a key step of ligustrazine to suppress the PMC functions. Besides, PPAR*γ* was regarded as a potential target for peritoneal fibrosis in either normoxic or hypoxic conditions, and the key step to abolish the changes of adhesion phenotype in fibroblast was to elevate PPAR*γ* level [44]. To determine the downstream effector of PPAR*γ* activation, we found that the transcription of HIF-1*α* inhibited by ligustrazine was negatively regulated by PPAR*γ*. In particular, HIF-1*α* is a pivotal regulator of hypoxia. We inferred that the activated PPAR*γ* could inhibit the transcription of HIF-1*α* in some dependent manner. It triggered us to explore the exact underlying mechanisms on how PPAR*γ* regulates HIF-1*α*, thereby influencing the ECM deposition and EMT change. Fortunately, based on the evidence [45, 46], coregulators SMRT or NCoR recruitment by PPAR*γ* was involved in regulating the expression of the downstream genes. HPMCs were transfected with si-SMRT and si-NCoR, respectively. Both the mRNA levels and protein expression of HIF-1*α* were significantly changed in the SMRT siRNA group rather than in the NCoR siRNA group. It indicated that SMRT was the crucial ligand in the regulatory mechanism of ligustrazine to activate PPAR*γ* on the suppression of HIF-1*α* transcription. Subsequently, we speculated that ligustrazine and PPAR*γ* might have some physical-binding manner. We predicted that ligustrazine could be docked successfully into several pocket residues to activate the receptor PPAR*γ*, evidenced by the molecular docking analysis. Among these binding sites, the residue Val 339/Ile 341 was critical for ligustrazine to activate PPAR*γ* by site-directed mutagenesis assay. And it is also reported that the conformational changes of PPAR*γ* can help the receptor to bind with dynamic ligand conformations [47]. This further confirmed that different bind sites were involved in the engagement of ligustrazine interaction with PPAR*γ*. All these findings indicated that PPAR*γ* is a direct target of ligustrazine, which provided a new perspective from the molecular aspects.

Importantly, we conducted PPA rodent models to validate our observations *in vivo*. However, pharmacokinetic studies reported that there were several disadvantages of ligustrazine on injured tissues, such as rapid absorption, fast metabolism, short half-time, low bioavailability, and poor tissue distribution [45, 46], which limited its biomedical application. To maintain an effective medication concentration, a controlled release preparation; that is, PLA nanoparticles loaded with ligustrazine were used on injury sites. It is well known that PLA as the lipophilic, biodegradable, and biocompatible polymer was widely reported to load with microparticles or nanoparticles [48–50]. The distinct physicochemical properties of the LN had good biocompatibility and surface activity. The novel agent could increase the drug particle solubility by nanotechnology, as evidence of high entrapment efficiency and loading capacity results. The results suggested that LN had site-specific drug delivery properties for preclinical application. The adhesion score/grade system showed a significant advantage of antiadhesion efficacy in the LN group. The findings indicated that LN was beneficial in preventing PPA. Masson staining further validated the advantage of LN in suppressing collagen fiber deposition objectively. Likewise, the results of the immunohistochemical analysis, Western blot, qPCR assay, and immunofluorescence analysis were all consistent with prior observations. All evidences suggested that LN may be regarded as an ideal approach for minimization of the adhesion effects.

## 5. Conclusion

**Figure 12:**
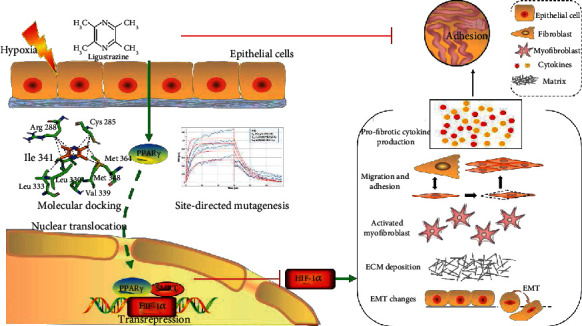
Ligustrazine activates PPAR*γ* on inhibition PMC functions through SMRT-dependent transrepression of HIF-1*α*.

In summary, our study uncovered that ligustrazine activated PPAR*γ* and interacted with PPAR*γ* in a specific site, following transrepression HIF-1*α* in an SMRT-mediated manner, thereby inhibiting hypoxia-induced PMC functions, ECM remodeling, and EMT phenotype changes (Figure 12). And we discovered a novel nanoparticle agent with sustained release properties, drug delivery efficiency, and good tissue penetration in rodent models. Our findings may provide new insights into PPA prevention either in preclinical or experimental research.

## Figures and Tables

**Table 1:** Animal grouping and treatment.

No.	Groups	Treatment
1	Sham	The cecum was exposed in the air for 5 min
2	Model	The cecum was scraped by dry gauze in the air until serosal petechiae appeared on the intestinal surfaces (lasting for 5 min)
3	Ligustrazine nanoparticles (LN)	The cecum was scraped by dry gauze in the air until serosal petechiae appeared on the intestinal surfaces (lasting for 5 min), and then, 5 ml/kg LN was applied to the abraded peritoneum and its surrounding areas before closing the peritoneal cavity
4	Sodium hyaluronate (SH)	The cecum was scraped by dry gauze in the air until serosal petechiae appeared on the intestinal surfaces (lasting for 5 min), and then, 0.5 ml/kg SH was applied to the abraded peritoneum and its surrounding areas before closing the peritoneal cavity
5	Polylactic acid (PLA)	The cecum was scraped by dry gauze in the air until serosal petechiae appeared on the intestinal surfaces (lasting for 5 min), and then, 0.5 ml/kg PLA was applied to the abraded peritoneum and its surrounding areas before closing the peritoneal cavity
6	Ligustrazine (LZ)	The cecum was scraped by dry gauze in the air until serosal petechiae appeared on the intestinal surfaces (lasting for 5 min), and then, 1 mg/ml LZ was applied to the abraded peritoneum and its surrounding areas before closing the peritoneal cavity

**Table 2:** Peritoneal adhesion scoring system [28].

Scores	Adhesion area	Description
0	None	None
1	0-25%	Thin, avascular, transparent
2	25-50%	Thin, avascular, transparent
3	50-75%	Thick, capillaries, opaque, sharp dissection required
4	75-100%	Thick, opaque, large vessels, sharp dissection required

## Data Availability

The data are available from the corresponding authors on reasonable request.

## Abbreviations

*α*-SMA: *α*-Smooth muscle actin
BLI: Biolayer interferometry
CTGF: Connective tissue growth factor
DMSO: Dimethyl sulfoxide
DAPI: Diamidino-phenyl-indole
DAB: Diaminobenzidine tetrahydrochloride
DAVID: Database for annotation, visualization, and integrated discovery
ELISA: Enzyme-linked immunosorbent assays
ECM: Extracellular matrix
EMT: Epithelial-mesenchymal transition
FDA: Fluorescein diacetate
FSP1: Fibroblast-specific protein 1
HE: Hematoxylin-eosin
HPMCs: Human primary peritoneal mesothelial cells
HIF-1*α*: Hypoxia-inducible factor-1*α*
ICAM-1: Intercellular cell adhesion molecular-1
LN: Ligustrazine nanoparticles
LZ: Ligustrazine
MMP2: Matrix metalloproteinases 2
MT: Mutant type
NcoR: Nuclear receptor coexpression
PLA: Polylactic acid
PPA: Postoperative peritoneal adhesion
PMCs: Peritoneal mesothelial cells
PBS: Phosphate-buffered saline
PPAR*γ*: Peroxisome proliferator-activated receptor *γ*
qRT-PCR: Quantitative real-time polymerase chain reaction
RPMCs: Rat primary peritoneal mesothelial cells
SMRT: Silencing mediator of retinoid and thyroid receptor
SH: Sodium hyaluronate
SD: Sprague-Dawley
TIMP-1: Tissue inhibitor of metalloproteinases-1
VEGF: Vascular endothelial growth factor
VCAM-1: Vascular cell adhesion molecule-1
WT: Wild type.

## Supplementary Materials

Supplementary Materials Additional supporting information may be found in the online version of this article. Supplementary Table S1: primers used for PCR amplification of wild-type and mutant-type PPAR*γ*. Supplementary Figures S1–S5: comparison of pET10 (WT) and pET11-pET15 genomic sequences. Supplementary Table S2: primers used for qRT-PCR.
