# Supplementary material for: Elucidating the Novel Mechanism of Ligustrazine in Preventing Postoperative Peritoneal Adhesion Formation
Source: Oxid Med Cell Longev. 2022 Mar 10;2022:9226022. doi: 10.1155/2022/9226022 (PMC8930249; doi:10.1155/2022/9226022)
Supplement: Supplementary Materials — Additional supporting information may be found in the online version of this article. Supplementary Table S1: primers used for PCR amplification of wild-type and mutant-type PPARγ. Supplementary Figures S1–S5: comparison of pET10 (WT) and pET11-pET15 genomic sequences. Supplementary Table S2: primers used for qRT-PCR. [file 9226022.f1.zip › Figure S1-5 Comparison of pET10 (WT) and pET11-pET15 genomic sequences.docx]

**Elucidating the novel mechanism of ligustrazine in** **preventing postoperative peritoneal adhesion formation**

Lili Yang^1,2,3^^,#^, Zhengjun Li^4,#^, Yao Chen^5^, Feiyan Chen^6^, Haopeng Sun^7^, Min Zhao^8^, Yanqi Chen^3^, Yali Wang^3^, Wenlin Li^1,2^, Li Zeng^1,2,^^3,^*, Yaoyao Bian^1,9,^*


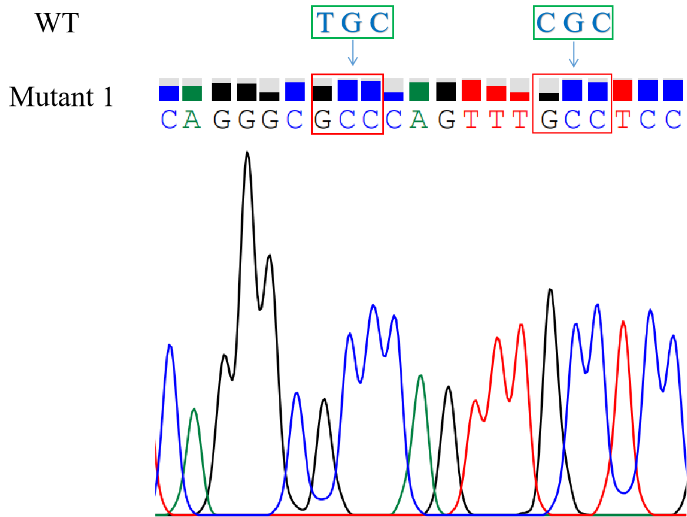


Supplementary Figure 1. Comparison of pET10 (WT) and pET11 (Mutant 1) genomic sequences shows the location of six base pair mutation in the pET11.


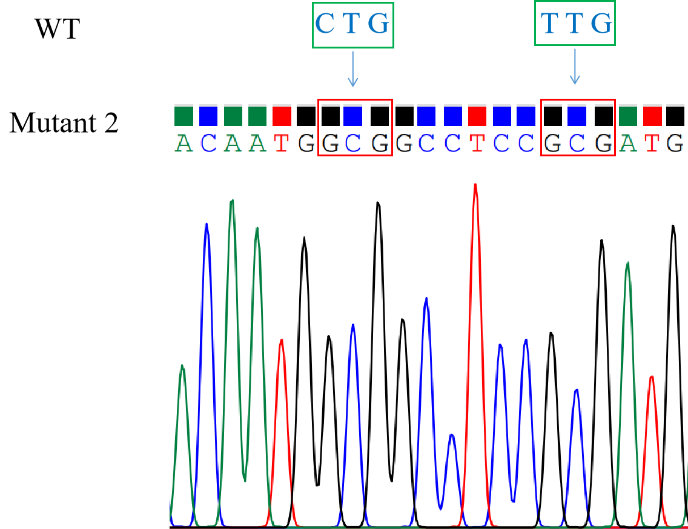


Supplementary Figure 2. Comparison of pET10 (WT) and pET12 (Mutant 2) genomic sequences shows the location of six base pair mutation in the pET12.


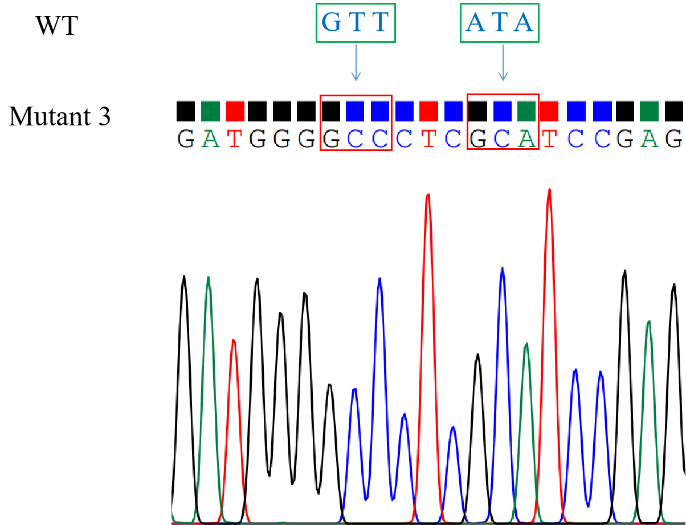


Supplementary Figure 3. Comparison of pET10 (WT) and pET13 (Mutant 3) genomic sequences shows the location of six base pair mutation in the pET13.


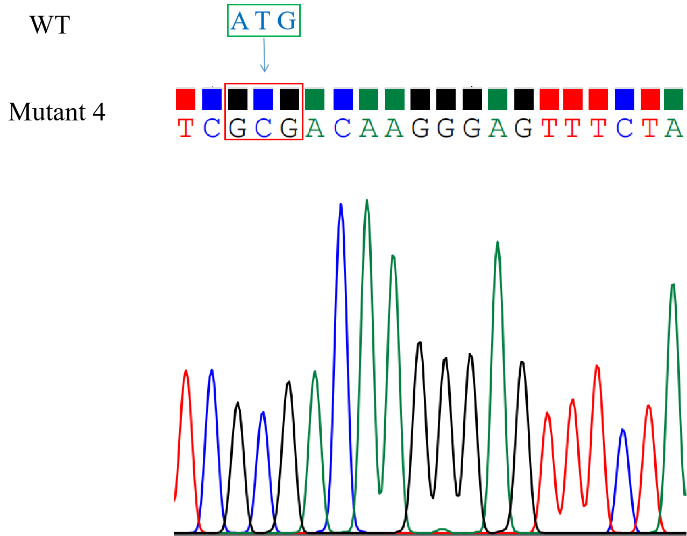


Supplementary Figure 4. Comparison of pET10 (WT) and pET14 (Mutant 4) genomic sequences shows the location of three base pair mutation in the pET14.


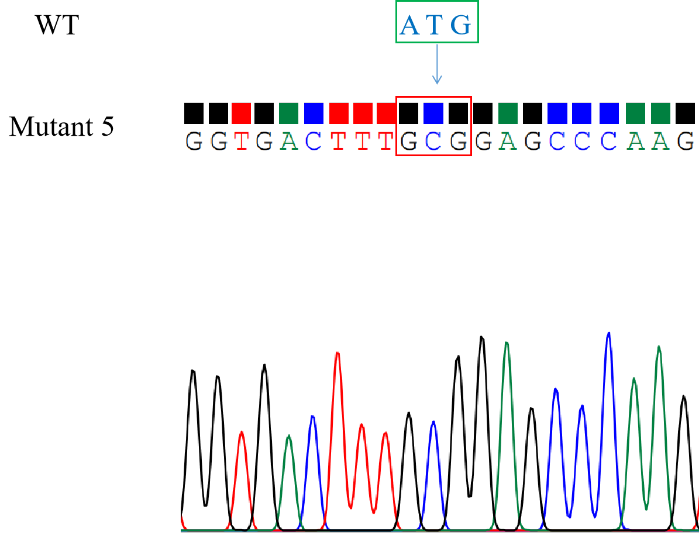


Supplementary Figure 5. Comparison of pET10 (WT) and pET15 (Mutant 5) genomic sequences shows the location of three base pair mutation in the pET15.
