# Supplementary material for: Elucidating the Novel Mechanism of Ligustrazine in Preventing Postoperative Peritoneal Adhesion Formation
Source: Oxid Med Cell Longev. 2022 Mar 10;2022:9226022. doi: 10.1155/2022/9226022 (PMC8930249; doi:10.1155/2022/9226022)
Supplement: Supplementary Materials — Additional supporting information may be found in the online version of this article. Supplementary Table S1: primers used for PCR amplification of wild-type and mutant-type PPARγ. Supplementary Figures S1–S5: comparison of pET10 (WT) and pET11-pET15 genomic sequences. Supplementary Table S2: primers used for qRT-PCR. [file 9226022.f1.zip › SUPPLEMENTARY DESCRIPTION.docx]

**SUPPLEMENTARY DESCRIPTION:**

**Supplementary materials**

Additional Supporting Information may be found in the online version of this article:

Supplementary Table S1∣Primers used for PCR amplification of wide-type and mutant PPARγ

Supplementary Fig S1-5∣Comparison of pET10 (WT) and pET11-pET15 genomic sequences

Supplementary Table S2∣Primers used for qRT-PCR
