# Supplementary material for: Elucidating the Novel Mechanism of Ligustrazine in Preventing Postoperative Peritoneal Adhesion Formation
Source: Oxid Med Cell Longev. 2022 Mar 10;2022:9226022. doi: 10.1155/2022/9226022 (PMC8930249; doi:10.1155/2022/9226022)
Supplement: Supplementary Materials — Additional supporting information may be found in the online version of this article. Supplementary Table S1: primers used for PCR amplification of wild-type and mutant-type PPARγ. Supplementary Figures S1–S5: comparison of pET10 (WT) and pET11-pET15 genomic sequences. Supplementary Table S2: primers used for qRT-PCR. [file 9226022.f1.zip › Table S1 Primers used for PCR amplification of wide-type and mutant PPAR_.docx]

**Table S1** **Primers used for PCR amplification of wide-type and mutant PPARγ**

| PPARγ | Primers | Sequence(5’-3’) |
| --- | --- | --- |
| Wide type | F | TAAGAAGGAGATATACCATGGGCATGGTTGACACAGAGATG |
|  | R | TGTCGACGGAGCTCGAATTCCTAGTGGTGGTGATGGTGATGGTACAAGTCCTTGTAGAT |
| Mutant 1 | F | TAAGAAGGAGATATACCATGGGCATGGTTGACACAGAGATG |
|  | Rm1 | GGAGGCAAACTGGGCGCCCTG |
|  | Fm1 | CAGGGCGCCCAGTTTGCCTCC |
|  | R | TGTCGACGGAGCTCGAATTCCTAGTGGTGGTGATGGTGATGGTACAAGTCCTTGTAGAT |
| Mutant 2 | F | TAAGAAGGAGATATACCATGGGCATGGTTGACACAGAGATG |
|  | Rm2 | CATCGCGGAGGCCGCCATTGT |
|  | Fm2 | ACAATGGCGGCCTCCGCGATG |
|  | R | TGTCGACGGAGCTCGAATTCCTAGTGGTGGTGATGGTGATGGTACAAGTCCTTGTAGAT |
| Mutant 3 | F | TAAGAAGGAGATATACCATGGGCATGGTTGACACAGAGATG |
|  | Rm3 | CTCGGATGCGAGGGCCCCATC |
|  | Fm3 | GATGGGGCCCTCGGATCCGAG |
|  | R | TGTCGACGGAGCTCGAATTCCTAGTGGTGGTGATGGTGATGGTACAAGTCCTTGTAGAT |
| Mutant 4 | F | TAAGAAGGAGATATACCATGGGCATGGTTGACACAGAGATG |
|  | Rm4 | CTCCCTTGTCGCGAAGCCTTG |
|  | Fm4 | CAAGGCTTCGCGACAAGGGAG |
|  | R | TGTCGACGGAGCTCGAATTCCTAGTGGTGGTGATGGTGATGGTACAAGTCCTTGTAGAT |
| Mutant 5 | F | TAAGAAGGAGATATACCATGGGCATGGTTGACACAGAGATG |
|  | Rm5 | CTTGGGCTCCGCAAAGTCACC |
|  | Fm5 | GGTGACTTTGCGGAGCCCAAG |
|  | R | TGTCGACGGAGCTCGAATTCCTAGTGGTGGTGATGGTGATGGTACAAGTCCTTGTAGAT |

“＿”Homology arm sites “ ” Restriction sites “ ”Mutation sequences
