# Supplementary material for: Elucidating the Novel Mechanism of Ligustrazine in Preventing Postoperative Peritoneal Adhesion Formation
Source: Oxid Med Cell Longev. 2022 Mar 10;2022:9226022. doi: 10.1155/2022/9226022 (PMC8930249; doi:10.1155/2022/9226022)
Supplement: Supplementary Materials — Additional supporting information may be found in the online version of this article. Supplementary Table S1: primers used for PCR amplification of wild-type and mutant-type PPARγ. Supplementary Figures S1–S5: comparison of pET10 (WT) and pET11-pET15 genomic sequences. Supplementary Table S2: primers used for qRT-PCR. [file 9226022.f1.zip › Table S2 Primers used for qRT-PCR.docx]

**Table S2** **Primers used for qRT-PCR**

| Species | Gene | Primer sequence (5’-3’) |
| --- | --- | --- |
| Rattus norvegicus | CTGF-Forward | GCCTGTTCCAAGACCTGT |
|  | CTGF-Reverse | GGATGCACTTTTTGCCCTTCTTA |
|  | VEGF-Forward | TGCCTCGTGGGACTGGAT |
|  | VEGF-Reverse | CCGGGCTTGGCGATTT |
|  | VCAM-1-Forward | CTACATCCACACTGACGCTGAG |
|  | VCAM-1-Reverse | CAGGGAATGAGTAGACCTCCACTT |
|  | ICAM-1-Forward | AAACGGGAGATGAATGGTACCTAC |
|  | ICAM-1-Reverse | TGCACGTCCCTGGTGATACTC |
|  | FSP1-Forward | AGGACAGACGAAGCTGCATT |
|  | FSP1-Reverse | CTCACAGCCAACATGGAAGA |
|  | Vinculin-Forward | CAAAGCAGAGTATTGCGAAGAA |
|  | Vinculin-Reverse | CATCACATAACTCAGCAATCTTCC |
|  | MMP2-Forward | CTGATAACCTGGATGCAGTCGT |
|  | MMP2-Reverse | CCAGCCAGTCCGATTTGA |
|  | TIMP-1-Forward | CATGGAGAGCCTCTGTGGAT |
|  | TIMP-1-Reverse | GTTCAGGCTTCAGCTTTTGC |
|  | E-cadherin-Forward | CAGGATTACAAGTTCCCGCCA |
|  | E-cadherin-Reverse | CACTGTCCGCTGCCTTCA |
|  | Cytokeratin 18-Forward | TCCTCAGCCATGTCTTCCTATG |
|  | Cytokeratin 18- Reverse | CTAAAACTTCCACCGCGT |
|  | Snail-Forward | GTCCTTGCTCCACAAACACCA |
|  | Snail-Reverse | CTGCCTTCCATCAGCCATCT |
|  | α-SMA-Forward | CATCAGGAACCTCGAGAAGC |
|  | α-SMA-Reverse | TCGGATACTTCAGGGTCAGG |
|  | HIF-1α-Forward | GATCAGCCAGCAAGTCCTTC |
|  | HIF-1α-Reverse | GGAGCTGTGAATGTGCTGTG |
|  | PPARγ-Forward | ATTCTGGCCCACCAACTTCGG |
|  | PPARγ-Reverse | TGGAAGCCTGATGCTTTATCCCCA |
|  | GAPDH-Forward | CATGCCGCCTGGAGAAACC |
|  | GAPDH-Reverse | GCCAGCCCCAGCATCAAAG |
| Homo sapiens | CTGF-Forward | AATGCTGCGAGGAGTGGGT |
|  | CTGF-Reverse | CGGCTCTAATCATAGTTGGGTCT |
|  | VEGF-Forward | GGAGGAGGGCAGAATCATCA |
|  | VEGF-Reverse | CTTGGTGAGGTTTGATCCGC |
|  | VCAM-1-Forward | CAAATCCTTGATACTGCTCATC |
|  | VCAM-1-Reverse | TTGACTTCTTGCTCACAGC |
|  | ICAM-1-Forward | ATACACACACACACACACGC |
|  | ICAM-1-Reverse | GCTATGCCTTGTCCTCTT |
|  | FSP1-Forward | GCTTCTTCTTTCTTGGTTTG |
|  | FSP1-Reverse | CTCCTTTAGTTCTGACTTGTTG |
|  | Vinculin-Forward | CTGAACCAGGCCAAAGGTT |
|  | Vinculin-Reverse | GATCTGTCTGATGGCCTGCT |
|  | MMP2-Forward | CAAAAACAAGAAGACATACAT |
|  | MMP2-Reverse | GCTTCCAAACTTCACGCTC |
|  | TIMP-1-Forward | TGGACTCTTGCACATCACTACCTGC |
|  | TIMP-1-Reverse | AGGCAAGGTGACGGGACTGGAA |
|  | E-cadherin-Forward | TCGACACCCGATTCAAAGTGG |
|  | E-cadherin-Reverse | TTCCAGAAACGGAGGCCTGAT |
|  | Cytokeratins 18-Forward | TCAGCAGATTGAGGAGAGCA |
|  | Cytokeratins 18-Reverse | TCTGACTCAAGGTGCAGCAG |
|  | Snail-Forward | GACTCCCAGACTCGCAAGG |
|  | Snail-Reverse | GACATGCGGGAGAAGGTTCG |
|  | α-SMA-Forward | GCGTGGCTATTCCTTGGTTA |
|  | α-SMA-Reverse | TGATGCTGTTGTAGGTGGTTTC |
|  | HIF-1α-Forward | ATACCAACAGTAACCAACCTC |
|  | HIF-1α-Reverse | CTGAATAATACCACTCACAAC |
|  | PPARγ-Forward | GGCTTCATGACAAGGGAGTTTC |
|  | PPARγ-Reverse | AACTCAAACTTGGGCTCCATAAAG |
|  | GAPDH-Forward | GGGGCTCTCCAGAACATCATCC |
|  | GAPDH-Reverse | ACGCCTGCTTCACCACCTTCTT |
